# Cuproptosis and Disulfidptosis Converge to Empower PD‐L1 Checkpoint Therapy via Cadict‐Induced PD‐L1 Translation

**DOI:** 10.1002/advs.202515367

**Published:** 2026-02-21

**Authors:** Shaoqing Huang, Shiyao Song, Xinhua Zhang, Jialing Gao, Ruihan Pu, Jiatong Dai, Xiaoxue Wu, Lulu Chen, Qinghai Li, Xiaoting Liu, Qi Zhou, Mei Song, Weiling He

**Affiliations:** ^1^ Center for Gastrointestinal Surgery The First Affiliated Hospital Sun Yat‐sen University Guangzhou Guangdong China; ^2^ Center for Hepato‐Pancreatico‐Biliary Surgery The First Affiliated Hospital Sun Yat‐sen University Guangzhou Guangdong China; ^3^ Department of Pediatrics The First Affiliated Hospital Sun Yat‐sen University Guangzhou Guangdong China; ^4^ Institute of Precision Medicine The First Affiliated Hospital Sun Yat‐Sen University Guangzhou Guangdong China; ^5^ School of Public Health Sun Yat‐sen University Guangzhou Guangdong China; ^6^ Department of Immunology Zhongshan School of Medicine Sun Yat‐sen University Guangzhou Guangdong China; ^7^ Department of General Surgery Hui Ya Hospital of The First Affiliated Hospital Sun Yat‐sen University Huizhou Guangdong China; ^8^ Department of Gastrointestinal Surgery School of Medicine Xiang'an Hospital of Xiamen University Xiamen University Xiamen Fujian China

**Keywords:** cancer, cuproptosis, disulfidptosis, integrated stress response (ISR), nanoparticles, PD‐L1

## Abstract

Immune checkpoint blockade (ICB) has emerged as a cornerstone of cancer therapy, yet its effectiveness remains restricted in PD‐L1‐low malignancies due to insufficient target expression. We herein develop the cuproptosis and disulfidptosis co‐delivery targeted (Cadict) nanodrug, an epidermal growth factor receptor (EGFR)‐targeted nanoplatform designed to co‐induce cuproptosis and disulfidptosis, thereby synergistically augmenting tumor cytotoxicity and sensitizing cancers to anti‐PD‐L1 therapy. Cadict exploits copper‐sulfur (Cu‐S) coordination chemistry to co‐deliver copper ions and cystine, while integrating glucose oxidase (GOx) to create a hypoglycemic milieu essential for disulfidptosis execution. This dual cytotoxic mechanism not only triggers immunogenic cell death‐like phenotype but also unexpectedly activates the integrated stress response (ISR), promoting PD‐L1 upregulation through Eif5b‐dependent translation. The resulting synergy between redox‐driven cytotoxicity and immune modulation potentiates anti‐PD‐L1 efficacy, leading to robust tumor regression and durable immunological memory. Our work presents a seminal strategy that leverages tumor redox vulnerabilities to advance cancer immunotherapy, providing a new paradigm for overcoming ICB resistance via targeted tumor sensitization.

## Graphical abstract

This study introduces Cadict, an EGFR‐targeted nanodrug that co‐delivers cuproptosis and disulfidptosis inducers to overcome immune resistance. Cadict synergistically enhances tumor cytotoxicity and sensitizes cancers to ICIs by upregulating PD‐L1 via an Eif5b‐dependent translation mechanism, fostering a potent antitumor immune response and establishing durable immunological memory. These findings demonstrate Cadict's therapeutic potential for immunologically cold and checkpoint‐refractory tumors.

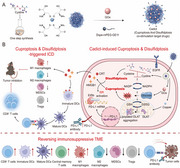

## 1 Introduction

Immune checkpoint blockade (ICB) therapies have revolutionized the cancer treatment landscape by unleashing host immune responses to eradicate tumors [1]. Central to this mechanism is the co‐inhibitory interaction of programmed cell death protein 1 (PD‐1) and its ligand PD‐L1, which suppresses cytotoxic T lymphocyte (CTL) activity, leading to their exhaustion and apoptosis and enabling immune evasion [2, 3]. Over 1000 clinical studies have demonstrated the therapeutic efficacy of PD‐1/PD‐L1 inhibitors, with approvals across multiple advanced malignancies like melanoma, non‐small cell lung cancer (NSCLC), microsatellite instable‐high (MSI‐H) or mismatch repair‐deficient (dMMR) colorectal cancer [4, 5, 6]. While PD‐L1 blockade agents such as atezolizumab and avelumab have demonstrated remarkable clinical efficacy, a substantial proportion of patients with low PD‐L1 expression fail to derive clinical benefit from these therapies [7]. Emerging evidence suggests that PD‐L1 upregulation may not only enhance chemotherapy responsiveness [8] but also potentiate targeted degradation of PD‐L1. These findings collectively imply that elevating PD‐L1 expression could broaden the therapeutic window for ICB, potentially sensitizing refractory patient populations to anti‐PD‐L1 therapies.

Copper upregulates PD‐L1 expression, thus facilitating tumor immune evasion [9]. Cuproptosis‐based nanomedicines have been shown to elevate tumor PD‐L1 levels and synergize with anti‐PD‐L1 therapy to enhance antitumor responses [10, 11]. A copper‐dependent form of regulated cell death facilitates immunogenic cell death (ICD) [12] while reprogramming the immunosuppressive tumor microenvironment (TME) to improve ICB efficacy [13, 14]. However, intrinsic or acquired resistance to single‐mode cell death often undermines the therapeutic benefit [15, 16]. Thus, identifying novel immunomodulatory cell death pathways and leveraging nonredundant mechanisms to enhance tumor immunogenicity represent a critical therapeutic frontier to overcome ICB resistance.

Disulfidptosis, a recently defined form of programmed cell death, is triggered by disulfide stress and NADPH (nicotinamide adenine dinucleotide phosphate) depletion under glucose deprivation [17, 18]. This process manifests aberrant accumulation of disulfide bonds in actin cytoskeletal proteins, leading to structural collapse and cell death. While emerging evidence suggests that disulfidptosis and its associated genes regulate immune function [19, 20, 21, 22, 23]. Its role in shaping the TME and potential for therapeutic synergy in combination with other cell death pathways remains poorly understood.

Nanomedicine has emerged as a transformative interdisciplinary field, revolutionizing disease diagnosis and treatment over recent years [24, 25]. In oncology, nanomedicines designed to induce single‐cell death pathways (e.g., ferroptosis, cuproptosis, or disulfidptosis) have demonstrated significant antitumor efficacy, both as monotherapies and in combination with ICB [26, 27, 28, 29, 30, 31, 32, 33]. Recent advances have enabled nanomedicines capable of co‐activating two distinct cell death modalities, including cuproptosis/ferroptosis, pyroptosis/ferroptosis, and ferroptosis/disulfidptosis combinations [34, 35, 36, 37, 38, 39, 40, 41, 42, 43]. However, no synthetic compounds have yet been developed to concurrently induce cuproptosis and disulfidptosis. Moreover, the interplay between this dual‐death approach, cellular redox homeostasis, and its immunoregulatory consequences remains unelucidated.

Notably, copper ions can coordinate with cystine to form nanocrystals stabilized by a quasi‐planar S‐Cu‐S configuration [44, 45, 46], and emerging preclinical evidence has validated the clinical translational potential of copper‐cystine coordination nanodrugs in murine and lapine models, highlighting their promise for bridging the gap from bench to bed. This foundational chemistry enables the rational design of Cu‐S coordination‐based nanomedicines capable of dually inducing cuproptosis and disulfidptosis.

**FIGURE 1:**
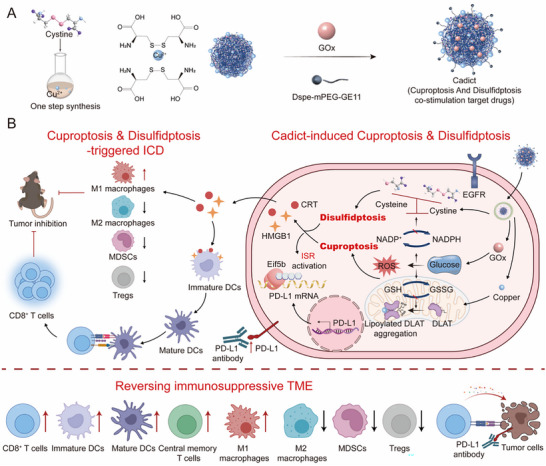
Schematic of Cadict for anti‐tumor therapy. A, B) One‐step symbiosis of Cadict (A) to induce cuproptosis and disulfidptosis, and its combination with PD‐L1 blockade to provoke robust immune activation, reprogram the immunosuppressive TME (B), and establish long‐term memory T cell‐mediated protection. glucose oxidase (GOx), nicotinamide adenine dinucleotide phosphate (NADPH), glutathione (GSH), glutathione disulfide (GSSG), epidermal growth factor receptor (EGFR), dihydrolipoamide S‐acetyltransferase (DLAT), ferredoxin (FDX1), lipoyl synthase (LIAS), immunogenic cell death (ICD), calreticulin (CRT), high mobility group box 1 (HMGB1), dendritic cells (DCs), myeloid‐derived suppressor cells (MDSCs), regulatory T cells (Tregs), tumor microenvironment (TME).

In this study, we present a dual cuproptosis/disulfidptosis strategy to enhance tumor cytotoxicity and upregulate PD‐L1 expression, thereby sensitizing tumors to anti‐PD‐L1 therapy. To implement this approach, we developed Cadict, an EGFR‐targeted nanodrug constructed through coordination‐driven self‐assembly of cystine and copper ions to co‐deliver inducers of both cell death modalities (Figure 1A). The nanoplatform was further engineered with GOx to create a hypoglycemic microenvironment essential for disulfidptosis. To confer tumor specificity and stability, Cadict was functionalized with the DSPE‐PEG‐GE11 conjugate. This design integrates three synergistic components: DSPE moiety provides a hydrophobic anchor for stable integration into the nanoplatform, PEG linker creates a stealth corona to prolong circulation and acts as a flexible spacer, and GE11 peptide enables active, EGFR‐mediated tumor targeting and internalization [47]. Once delivered to the tumor site, Cadict initiated both cuproptosis and disulfidptosis in vivo, manifesting features of ICD and promoting antitumor immunity. Unexpectedly, it also activated the ISR, driving tumoral PD‐L1 upregulation via Eif5b‐dependent translation. This dual mechanism markedly potentiated anti‐PD‐L1 therapy, resulting in robust tumor suppression and durable immunological memory in tumor models (Figure 1B). Furthermore, our study proposes a novel therapeutic strategy to enhance anti‐PD‐L1 efficacy through targeted PD‐L1 upregulation, thereby overcoming intrinsic resistance in low PD‐L1‐expressing tumors.

## 2 Results

### 2.1 Dual Induction of Cuproptosis and Disulfidptosis Synergistically Enhances Tumor Suppression and Potentiates ICB Efficacy

**FIGURE 2:**
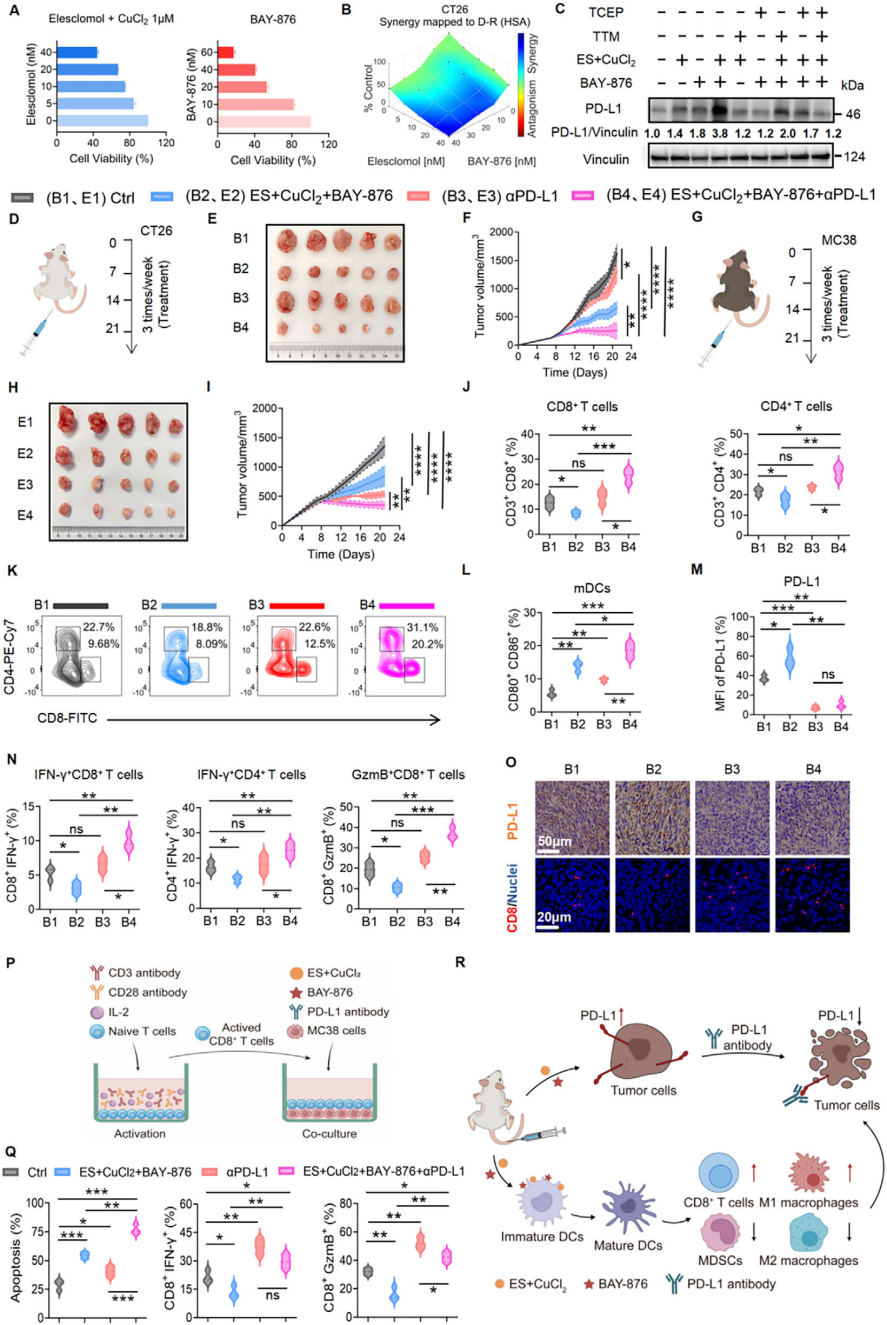
Induction of cuproptosis and disulfidptosis drives potent tumor suppression and ICB sensitization. A) Viability of CT26 cells treated with Elesclomol (ES‐0, 5, 10, 20, 40 nm) + CuCl_2_ (1 µm) or BAY‐876 (0, 10, 20, 40, 60 nm). B) Clonogenic survival of CT26 cells treated with vehicle, ES+CuCl_2_, BAY‐876, or their combination (n = 3 independent experiments). Colonies were quantified after 10 days (ImageJ v1.53c). Drug synergy was analyzed using Combenefit (blue: synergy; red: antagonism). C) PD‐L1 expression in MC38 cells pretreated with TTM (20 µm) or TCEP (1 mm) for 12 h, followed by exposure to ES+CuCl_2_ (20 nm + 1 µm) or BAY‐876 (20 nm) for 36 h. Protein levels were analyzed by Western blot and normalized to Vinculin. D) Treatment schedule in the subcutaneous CT26 tumor model. E, F) Representative images (E) and statistical analysis of tumor volumes (F) in BALB/c mice bearing CT26 tumors (B1, Ctrl; B2, ES+CuCl_2_+BAY‐876; B3, αPD‐L1; B4, ES+CuCl_2_+BAY‐876+αPD‐L1). G) Treatment schedule in the subcutaneous MC38 tumor model. H, I) Representative images (H) and statistical analysis of tumor volumes (I) in C57BL/6 mice bearing MC38 tumors (E1, Ctrl; E2, ES+CuCl_2_+BAY‐876; E3, αPD‐L1; E4, ES+CuCl_2_+BAY‐876+αPD‐L1). J, K) Representative flow cytometry plots (K) and quantification (J) of tumor‐infiltrating CD3^+^CD8^+^ and CD3^+^CD4^+^ T cells in CT26 tumors (n = 3). L) The percentages of matured DCs (CD80^+^CD86^+^) populations in CT26 tumors (n = 3). M) Quantification of PD‐L1 expression in treated CT26 tumors (n = 3). N) The percentages of IFN‐γ^+^CD8^+^, IFN‐γ^+^CD4^+^, and GzmB^+^CD8^+^ T cell populations in CT26 tumors under various treatments (n = 3). O) Representative images of immunofluorescence staining of CD8^+^ T cells and immunohistochemistry staining of PD‐L1 protein in the tumor sections. P) Schematic of CD8^+^ T cell‐MC38 coculture model: Splenic CD8^+^ T cells (C57BL/6) were activated with anti‐CD3 (5 mg ml^−1^)/anti‐CD28 (1 mg ml^−1^)/IL‐2 (50 U ml^−1^), and cocultured with MC38 cells in the presence of ES+CuCl_2_, BAY‐876, and anti‐PD‐L1. Q) Quantification of MC38 cell apoptosis rates and the proportions of IFN‐γ^+^CD8^+^ or GzmB^+^CD8^+^ T cell populations in 1:1 cocultures under various treatments. R) Schematic diagram illustrating the proposed mechanism of combination therapy‐enhanced antitumor immunotherapy. Data are presented as mean ± SD, with two‐way analysis of variance (ANOVA) test (F, I) or one‐way ANOVA test (J, L‐N, Q). ns, non‐significant, ^*^ *p* < 0.05, ^**^ *p* < 0.01, ^***^ *p* < 0.001, ^****^ *p* < 0.0001.

To assess whether combined induction of cuproptosis and disulfidptosis enhances cytotoxicity and antitumor immunity, we treated microsatellite‐stable (MSS) CT26 and MSI‐H MC38 colorectal cancer (CRC) cells with the cuproptosis inducer CuCl_2–_Elesclomol (Cu‐ES) and the disulfidptosis inducer BAY‐876. In vitro, either Cu‐ES or BAY‐876 reduced cell viability, while their combination synergistically enhanced cell death in both cell lines (Figure 2A,B; Figure S1A,B). Dual treatment also upregulated PD‐L1 expression (3.8‐fold vs. untreated controls), an effect reversed by the cuproptosis inhibitor TTM or the disulfidptosis inhibitor TCEP, linking PD‐L1 induction to both cell death modes (Figure 2C). In vivo, Cu‐ES + BAY‐876 regimen suppressed tumor growth in syngeneic CT26 and MC38 models and further boosted anti‐PD‐L1 efficacy (Figure 2D–I; Figure S1C–F). This triple combination (Cu‐ES+BAY‐876+anti‐PD‐L1) remodeled the TME, increased CD4^+^/CD8^+^ T cell infiltration and mature dendritic cells (mDCs) while reducing tumoral PD‐L1 levels (Figure 2J–M; Figure S1G–J). Importantly, it overcame intrinsic anti‐PD‐L1 resistance and reversed T cell suppression in CT26 and MC38 tumors by expanding functional T cell subsets (IFN‐γ^+^CD8^+^ T, IFN‐γ^+^CD4^+^ T, and GzmB^+^CD8^+^ T cells), reducing MDSCs infiltration, and increasing the M1/M2 macrophage ratio (Figure 2N,O; Figure S1K–P). These results demonstrate that dual cuproptosis/disulfidptosis induction can potentiate ICB efficacy even in MSS CRC that is otherwise resistant to ICB therapy [48].

Paradoxically, despite robust tumor suppression, Cu‐ES+BAY‐876 treatment resulted in markedly reduced infiltration of total CD4^+^/CD8^+^ T cells and effector T cell subsets compared to controls (Figure 2J,N; Figure S1H,K). This immunosuppressive shift coincided with a dramatic PD‐L1 upregulation, an effect reversed by anti‐PD‐L1 therapy. To further investigate whether PD‐L1 induction impaired T cell function, we co‐cultured splenic CD8^+^ T cells with MC38 cells (Figure 2P). Consistent with in vivo findings, Cu‐ES+BAY‐876‐treated tumor cells remained susceptible to lysis but suppressed IFN‐γ and Granzyme B production in CD8^+^ T cells, a phenomenon relieved by anti‐PD‐L1 blockade (Figure 2Q). Thus, dual cuproptosis/disulfidptosis induction not only exerts potent cytotoxicity but also drives PD‐L1 upregulation, sensitizing tumors to anti‐PD‐L1 therapy (Figure 2R).

### 2.2 Synthesis and Characterization of the Cuproptosis and Disulfidptosis Co‐Delivery Targeted (Cadict) Nanodrug

**FIGURE 3:**
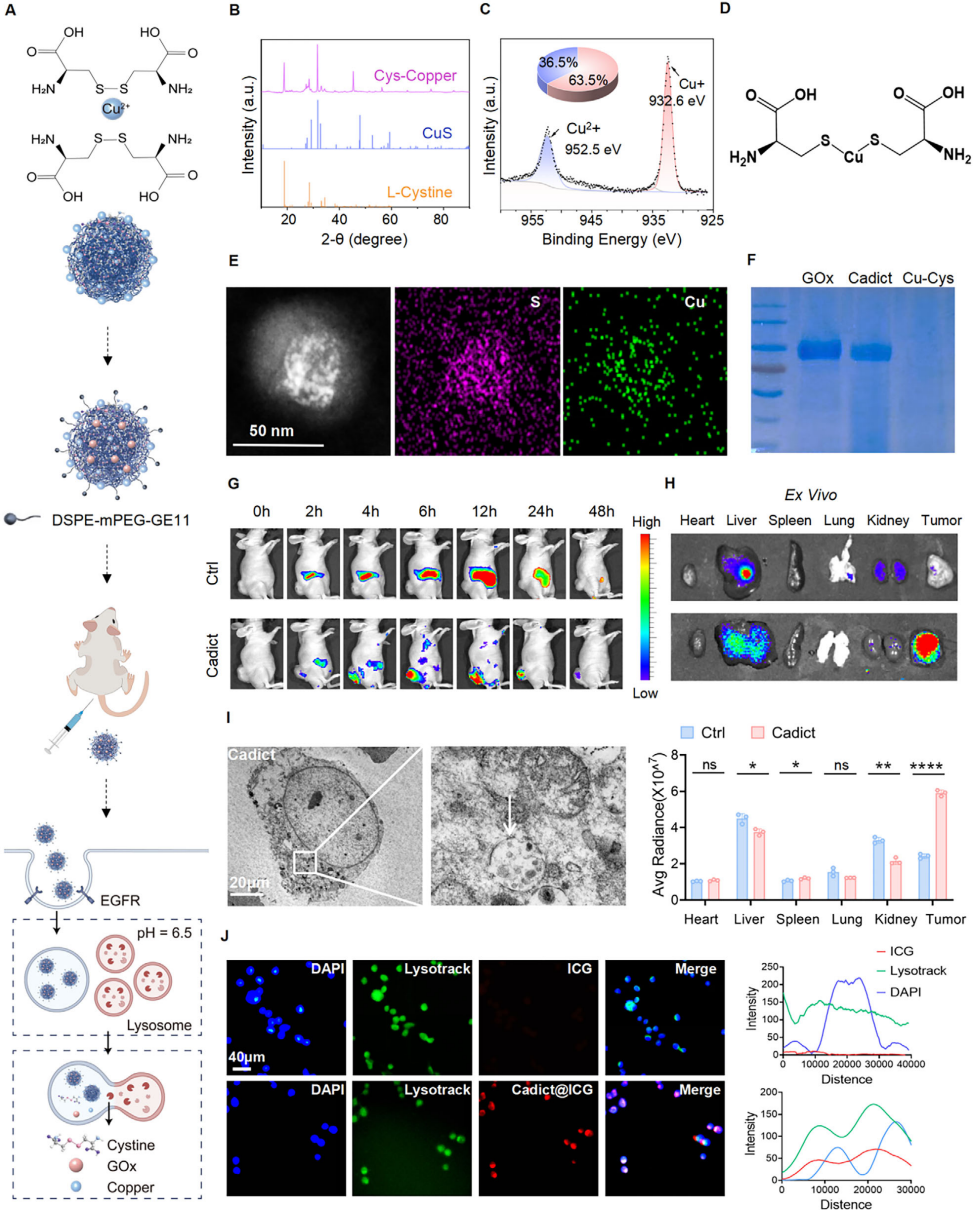
Synthesis and characterization of Cadict. A) Illustration of cystine and copper co‐coordination structure. B) XRD patterns of the Cystine Copper copolymer (Cys‐Cu), Cystine, and CuS. C) XPS spectra of Cu 2p in Cystine Copper copolymer. D) Illustration of the S‐Cu‐S structure in Cystine Copper copolymer. E) TEM image of Cadict and corresponding elemental mappings of Cu and S signals in water. F) Coomassie Blue analysis of GOx (lane 1), Cadict (lane 2), and Cystine Copper copolymer (lane 3). G) Fluorescence imaging of the subcutaneous MC38 tumors in mice receiving i.v. injection of ICG (Ctrl) or ICG‐labeled Cadict at the indicated times. The color bar represents the corresponding fluorescence intensity. H) Representative fluorescence images and average fluorescence intensities of main organs and tumors at 12 h post *i.v*. injection of ICG (Ctrl) or ICG‐labeled Cadict. I) Representative Bio‐TEM images of MC38 cells after incubation with Cadict (2 µm) for 2 h. White arrows show endocytic Cadict. Scale bars: 20 µm. J) Intracellular trafficking of ICG‐labeled Cadict (red) in MC38 cells at 4 h. Nuclei (blue) and lysosomes (green) shown. Fluorescence intensity profiles (right). Scale bars, 40 µm. Data are presented as mean ± SD, with an unpaired two‐tailed Student's *t*‐test (H). ns, non‐significant, ^*^ *p* < 0.05, ^**^ *p* < 0.01, ^****^ *p* < 0.0001.

Cuproptosis is driven by intracellular copper ion accumulation, while disulfidptosis primarily results from cystine overload. To co‐deliver copper ions and cystine into tumors for simultaneous induction of both cell death, we developed Cadict—a targeted co‐delivery nanodrug synthesized via copper‐disulfide coordination self‐assembly (Figure 3A; Figure S2A). Whether cystine was added dropwise to copper ions or vice versa, the resulting nanoparticles exhibited a uniform size of 5–10 nm (Figure S2B). X‐ray diffraction (XRD) confirmed a crystalline structure with characteristic peaks corresponding to cystine (JCPDS: 37–1802) and CuS (JCPDS: 79–2321) [46], indicating coexistence of cystine and copper (Figure 3B). X‐ray photoelectron spectroscopy (XPS) revealed that 63.5% of copper ions existed as Cu^+^, suggesting that cystine's reducing properties promote Cu^+^‐cystine coordination during synthesis (Figure 3C). This aligned with previous reports of S‐Cu‐S coordination structures (Figure 3D) [49]. Notably, Cu^+^ ions induce cuproptosis more effectively than Cu^2+^ [50]. Transmission electron microscopy (TEM) elemental mapping verified the presence of Cu and S within the coordination framework (Figure 3E), with observed particle sizes ranging from 10–20 nm. These particles are thus capable of co‐delivering Cu^+^, Cu^2+^, and cystine.

To amplify disulfidptosis, which requires both cystine accumulation and glucose deprivation [17], we incorporated GOx during coordination to deplete intratumoral glucose. Titration experiments revealed that 10 mg of GOx yielded particles with optimal size and a polydispersity index (PDI) below 0.3 (Figure S2C). To further enhance tumor targeting, we functionalized the nanoparticles with an EGFR‐targeting peptide (GE11) and designated the final formulation as Cadict. Coomassie blue staining confirmed GOx (∼100 kDa) incorporation into Cadict (Figure 3F). TEM revealed uniform nanoparticle morphology (Figure S2D), with a diameter of 11.7 nm, PDI = 0.294 (Figure S2E), a zeta potential of +21.4 mV (Figure S2F), and stability in PBS up to 6 days (Figure S2G). Collectively, these data demonstrate the successful synthesis of tumor‐targeting nanoparticles with favorable physicochemical properties.

Successful targeted delivery of Cadict is a prerequisite for unlocking its therapeutic potential. To evaluate tumor‐targeting efficacy in vivo, we intravenously administered ICG‐labeled Cadict to MC38 tumor‐bearing mice. IVIS imaging revealed progressive tumor accumulation, peaking at 12 h and persisting through 48 h, whereas free ICG (Ctrl) primarily accumulated in the livers and kidneys, with complete clearance by 48 h. Ex vivo imaging confirmed a 3‐fold higher tumor fluorescence in Cadict‐treated mice compared to controls (Figure 3G,H). Following successful tumor targeting, the next pivotal step is to achieve intratumoral drug release [51]. Bio‐TEM imaging revealed Cadict's efficient endocytic uptake into tumor cells (Figure 3I), consistent with established nanomaterial internalization pathways, where endocytic uptake precedes lysosomal trafficking for intracellular drug release [52]. To further assess intracellular trafficking, we treated MC38 cells with ICG‐labeled Cadict and observed strong co‐localization with LysoTracker‐stained lysosomes, in contrast to free ICG drugs (Figure 3J). In vitro, Cadict was efficiently internalized and accumulated in MC38 cells, reaching a plateau at approximately 12 h. In contrast, the GE11‐unconjugated control showed only marginal intracellular accumulation even after 24 h of incubation (Figure S2H), confirming that GE11 conjugation confers specific and potent targeting capability. ICP‐MS analysis revealed that Cadict treatment induced a 14.3‐fold increase in intracellular copper accumulation in MC38 cells, confirming its effective delivery and release capability (Figure S2I). Cadict demonstrated favorable in vivo pharmacokinetic properties, featuring a plasma half‐life of ∼1.22 h (Figure S2J). Under acidic conditions (pH ≈5.6) typical of lysosomes and TME [53, 54], Cadict's Cu‐S coordination dissociated, enabling efficient drug release. In summary, Cadict nanoparticles achieve effective tumor targeting, cellular internalization, and spatiotemporal drug release for dual cuproptosis and disulfidptosis induction.

### 2.3 Cadict Provokes Cuproptosis and Disulfidptosis in Tumor Cells

**FIGURE 4:**
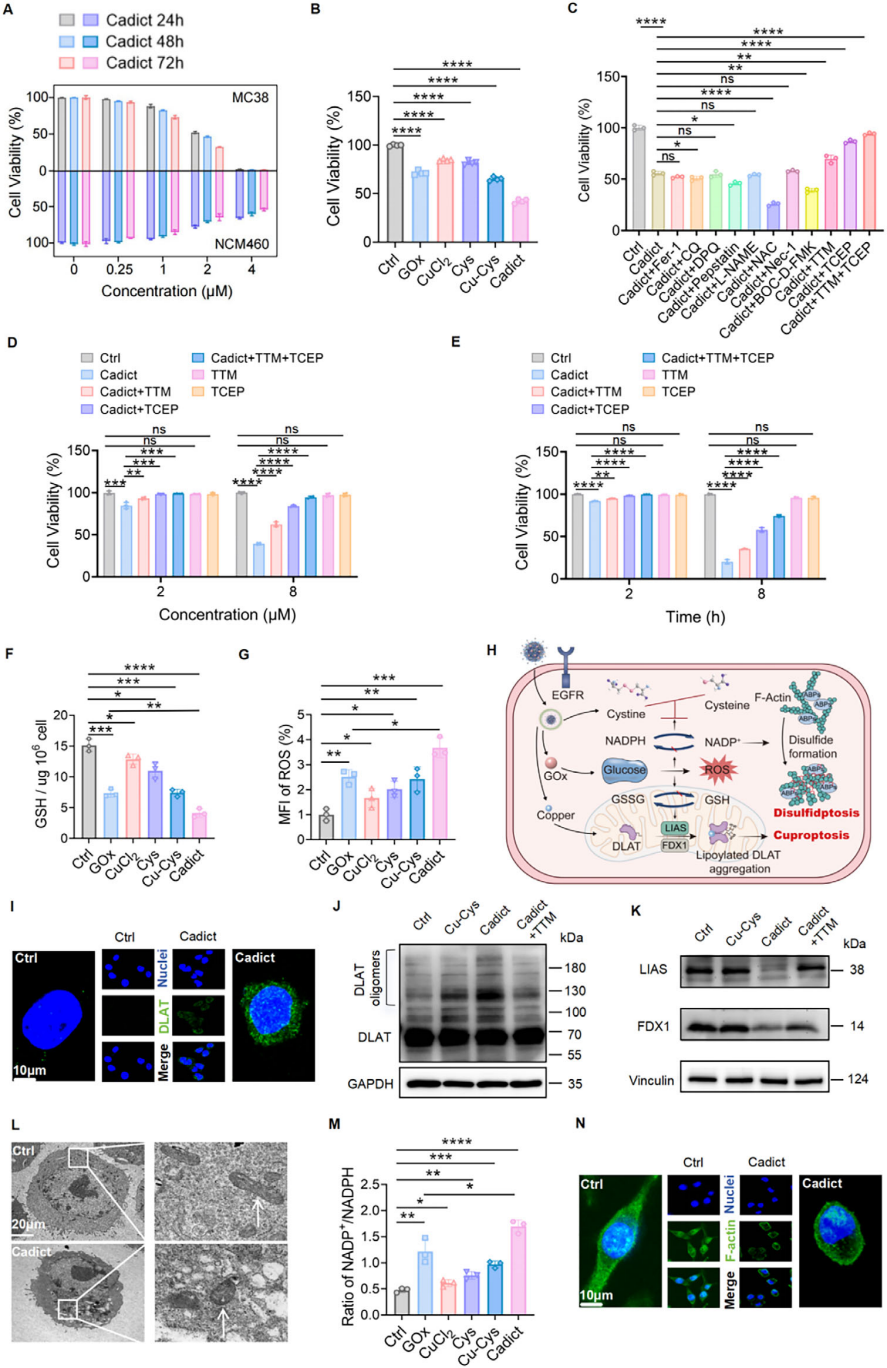
Cadict triggers dual cuproptosis and disulfidptosis in tumor cells. A) Viability of MC38 CRC cells and normal colon epithelial cells (NCM460) treated with Cadict (0, 0.125, 0.25, 0.5, 1.0, 2.0, 4.0 µm) for 24, 48, and 72 h. B) Viability of MC38 cells treated with PBS, GOx (0.2 µg ml^−1^), CuCl_2_ (0.2 µg ml^−1^), Cys (0.2 µg ml^−1^), Cu‐Cys (2 µm), Cadict (2 µm) for 24 h. C) Viability of MC38 cells pretreated with Fer‐1 (10 µm), CQ (50 µm), DPQ (10 µm), Pepstatin (1 µm), L‐NAME (300 µm), NAC (1 mm), Nec‐1 (20 µm), D‐BOC‐FMK (50 µm), TTM (20 µm), TCEP (1 mm) for 12 h, followed by Cadict (2 µm) for 24 h. D, E) Viability of MC38 cells pretreated with 20 µm TTM and/or 1 mm TCEP for 12 h, followed by treatment with different concentrations of Cadict (2 and 8 µm) for 4 h (D), or at different time points (2 and 8 h) with Cadict (4 µm) (E). F, G) Quantification of intracellular GSH (F) and ROS (G) levels in MC38 cells across treatments. H) Schematic diagram illustrating cuproptosis‐disulfidptosis synergy in cell death induction. I) Confocal laser scanning microscope (CLSM) images of DLAT in Cadict (2 µm, 4 h) treated MC38 cells. J, K) Western blot analysis of DLAT oligomers (J), FDX1 and LIAS (K) expression in MC38 cells treated with PBS, Cu‐Cys (2 µm), Cadict (2 µm), or TTM (20 µm) for 24 h. L) Bio‐TEM images showing mitochondria (boxed) in Cadict (2 µm, 4 h) treated MC38 cells. M) Quantification of intracellular NADP^+^/NADPH ratios in MC38 cells across treatments. N) CLSM images of F‐actin in Cadict (2 µm, 4 h) treated MC38 cells. Data are presented as mean ± SD, with one‐way ANOVA test (B, C, F, G, M) or two‐way ANOVA test (D, E). ns, non‐significant, ^*^ *p* < 0.05, ^**^ *p* < 0.01, ^***^ *p* < 0.001, ^****^ *p* < 0.0001.

Cadict exhibited dose‐ and time‐dependent cytotoxicity against tumor cells, achieving complete cell elimination at 4 µm (Figure 4A; Figure S3A), while causing negligible toxicity to non‐tumor cells, confirming its favorable biocompatibility (Figure 4A). Superior efficacy over individual components was further validated through cell viability, colony formation, transwell, live/dead staining, and apoptosis assays (Figure 4B; Figure S3B–E).

To delineate the mechanism of Cadict‐induced cell death, we systematically blocked major death pathways using pharmacologic inhibitors targeting apoptosis (BOC‐D‐FMK), necroptosis (necrostatin‐1), ferroptosis (ferrostatin‐1), autophagy (chloroquine), pyroptosis (disulfiram), and oxidative stress (N‐acetyl cysteine). None of these inhibitors attenuated Cadict's cytotoxicity. In contrast, both the cuproptosis inhibitor TTM and the disulfidptosis inhibitor TCEP significantly restored cell viability, and the co‐administration of TTM and TCEP restored cell viability to near‐control levels, strongly supporting that Cadict‐induced cell death is mediated through the concurrent activation of these two pathways (Figure 4C). Notably, TTM and TCEP efficiently rescued cell survival even under escalated Cadict doses (up to 8 µm) and prolonged exposure (8 h), demonstrating synergistic efficacy (Figure 4D,E). Mechanistically, cystine overload impairs glutathione (GSH) synthesis, while excess copper ions consume GSH, a key thio‐containing copper chelator [14]. Cadict treatment markedly reduced intracellular GSH levels by ∼70% compared to controls, and caused a significantly greater, synergistic depletion (GOx vs. Cadict, *p* = 0.004), implying its potent GSH‐scavenging capability (Figure 4F). This GSH exhaustion rendered cells vulnerable to oxidative damage, culminating in cell death. While CuCl_2_, cystine (Cys), or CuCl_2_‐cystine (Cu‐Cys) moderately elevated ROS (∼2‐fold), Cadict treatment synergistically amplified ROS accumulation (∼4‐fold) (Figure 4G). Cuproptosis is characterized by copper‐dependent aggregation of lipoylated dihydrolipoamide S‐acetyltransferase (DLAT), a pivotal TCA cycle enzyme [55]. Copper binding destabilizes Fe‐S cluster proteins, such as ferredoxin (FDX1) and lipoyl synthase (LIAS), which are essential for DLAT lipoylation (Figure 4H) [56]. As expected, Cadict fostered DLAT aggregation, visualized as DLAT puncta (Figure 4I), and downregulated FDX1 and LIAS levels, an effect reversed by TTM, confirming that Cadict elicits cell death through cuproptosis (Figure 4J,K). TEM further revealed cuproptosis‐associated mitochondrial damage following Cadict treatment, manifested as shrunken mitochondria with fragmented cristae and elevated membrane density (Figure 4L). In parallel, GOx depletes intracellular glucose, limiting NADPH production, a key redundant factor required to counteract disulfide stress [17]. We observed an elevated NADP^+^/NADPH ratio in tumor cells treated with GOx or Cadict (Figure 4M), which triggered cystine accumulation and F‐actin contraction with detachment from the plasma membrane, supporting the onset of disulfidptosis (Figure 4N). Taken together, these findings demonstrate that Cadict evokes both cuproptosis and disulfidptosis in tumor cells.

### 2.4 Cadict Elicits ICD‐Like Effects and Activates Antitumor Immune Response In Vivo

**FIGURE 5:**
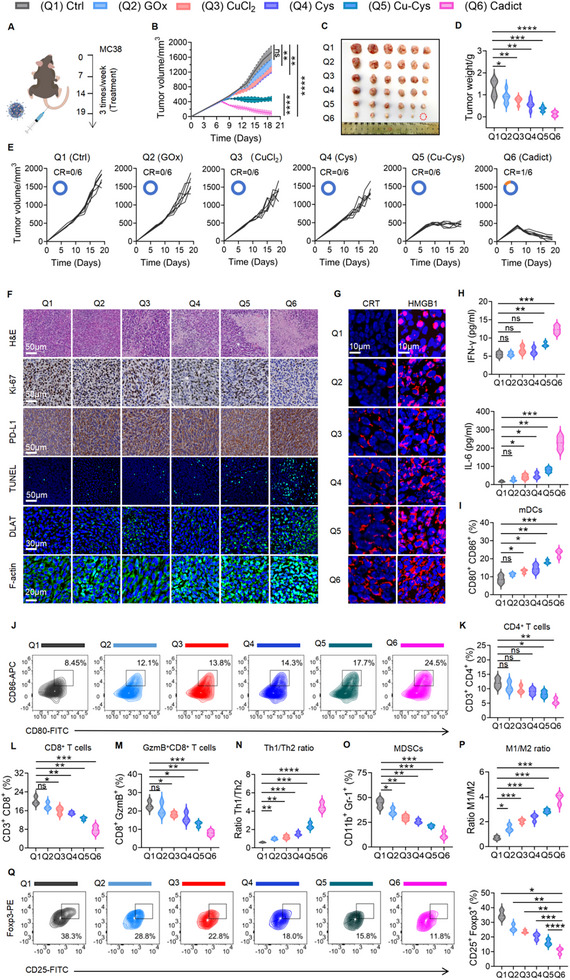
Cadict elicits ICD‐like effects and activates antitumor immunity in vivo. A) Treatment schedule in the subcutaneous MC38 tumor model. B, E) Quantification of tumor volumes (B) and tumor growth kinetics (E) across treatment groups [complete response: CR] (n = 6). C, D) Representative images (C) and tumor weight statistics (D) of excised MC38 across treatments (Q1, Ctrl; Q2, GOx; Q3, CuCl_2_; Q4, Cys; Q5, Cu‐Cys; Q6, Cadict) (n = 6). F, G) Representative images of H&E, TUNEL, immunohistochemistry staining of Ki‐67, PD‐L1, immunofluorescence staining of DLAT, F‐actin, CD8^+^ T cells (F), and HMGB1/CRT (G) in tumor sections. H) Serum IFN‐γ and IL‐6 levels in MC38 tumor‐bearing mice with different treatments (n = 6). I, J) Representative flow cytometry plots (J) and quantification (I) of tumor‐infiltrating matured DCs (CD80^+^CD86^+^) in MC38 tumors (n = 3). K–M) The percentages of CD4^+^ T (K), CD8^+^ T (L), and GzmB^+^CD8^+^ T (M) cell populations in MC38 tumors (n = 3). N‐P) The percentages of MDSCs (CD11b^+^Gr‐1^+^) (O), Th1/Th2 ratios (N), and M1/M2 macrophage ratios (P) in MC38 tumors across treatments (n = 3). Q) Representative flow cytometry plots and quantification of tumor‐infiltrating Tregs (CD25^+^Foxp3^+^) in MC38 tumors (n = 3). Data are presented as mean ± SD, with a two‐way ANOVA test (B) or one‐way ANOVA test (D, H, I, K–Q). ns, non‐significant, ^*^ *p* < 0.05, ^**^ *p* < 0.01, ^***^ *p* < 0.001, ^****^ *p* < 0.0001.

To evaluate the therapeutic potential of Cadict in vivo, we treated MC38 tumor‐bearing mice with control (Ctrl), GOx, CuCl_2_, Cys, Cu‐Cys, or Cadict. Cadict displayed dose‐dependent tumor suppression, achieving near‐complete tumor eradication at 9 mg kg^−1^ (Figure S4A–D). However, higher doses (≥7 mg kg^−1^) caused transient weight loss (Figure S4E,F). To balance efficacy and tolerability, we selected 5 mg kg^−1^ Cadict for follow‐up studies. Both Cu‐Cys and Cadict treatments markedly reduced tumor size and weight compared to other groups (Figure 5A–D), with Cadict demonstrating superior efficacy and achieving complete tumor regression in one mouse by day 18 (Figure 5E). Histopathological analysis revealed extensive tumor necrosis and nucleolysis, reduced proliferative activity (Ki67), and enhanced cell death (TUNEL staining) in Cadict‐treated tumors, suggesting that Cadict possesses the most superior anti‐tumor effect. Additionally, Cadict treatment induced DLAT aggregation and F‐actin contraction within tumor tissues, implying concurrent activation of cuproptosis and disulfidptosis in vivo (Figure 5F). Moreover, consistent evidence from both in vitro and in vivo experiments demonstrated that Cadict elicited the highest exposure of calreticulin (CRT) and release of high mobility group box 1 (HMGB1)—hallmark damage‐associated molecular patterns (DAMPs) of ICD—compared to other treatments (Figure 5G; Figure S4G). These findings suggest that Cadict‐mediated dual induction of cuproptosis and disulfidptosis triggers robust ICD‐like effects and antitumor immunity in vivo.

To investigate Cadict's potential to elicit anticancer immunity, we analyzed immune cell composition in the serum, spleens, and tumors of MC38 tumor‐bearing mice across treatment groups. Cadict treatment elevated serum levels of IFN‐γ (2‐3‐fold) and IL‐6 (∼12‐fold) by compared to controls, indicating intensified systemic inflammation (Figure 5H). It also increased the percentages of circulating DCs (CD11c^+^MHC‐II^+^), CD4^+^ T cells (CD3^+^CD4^+^), and CD8^+^ T cells (CD3^+^CD8^+^) (Figure S4H–J), while promoting expansion of splenic DCs and NK cells (CD45^+^NK1.1^+^CD3^−^) (Figure S4K,L). Despite enhanced systemic immunity and increased matured DCs (CD80^+^CD86^+^) infiltration into the TME (Figure 5I,J), the frequencies of CD4^+^ T, CD8^+^ T, and GzmB^+^CD8^+^ T cells were less than half of those observed in controls (Figure 5K–M). This reduction likely reflects elevated intratumoral PD‐L1 expression, which promotes T cell apoptosis and exhaustion. Furthermore, we observed elevated M1/M2 and Th1/Th2 ratios, as well as a decrease in immunosuppressive MDSCs (CD11b^+^Gr‐1^+^) and regulatory T cells (CD25^+^Foxp3^+^, Tregs) across GOx, CuCl_2_, Cys, Cu‐Cys‐treated groups. These pro‐inflammatory shifts were most pronounced following Cadict treatment (Figure 5N–Q; Figure S4M).

We assessed in vivo biosafety by measuring hepatic, renal, and cardiac function markers, including alanine aminotransferase (ALT), blood urea nitrogen (BUN), UREA, and lactate dehydrogenase (LDH). All markers remained stable across treatment groups (Figure S4N). Serum aspartate aminotransferase (AST) levels in Cadict‐treated mice were significantly reduced compared to controls yet remained within the physiological range for mice (36.31–235.48 U L^−1^). Histological analysis revealed no tissue damage or morphological abnormalities in major organs (heart, liver, spleen, lung, and kidney) post Cadict administration (Figure S4O). Body weights remained stable throughout (Figure S4P). Moreover, Cadict exhibited exceptional hemocompatibility, with hemolysis rates below 5% across all tested concentrations (Figure S4Q). Altogether, these data demonstrate that Cadict activates systemic antitumor immunity without detrimental effects on normal tissues.

### 2.5 Cadict Elevates PD‐L1 Expression through Eif5b‐Dependent Translation

**FIGURE 6:**
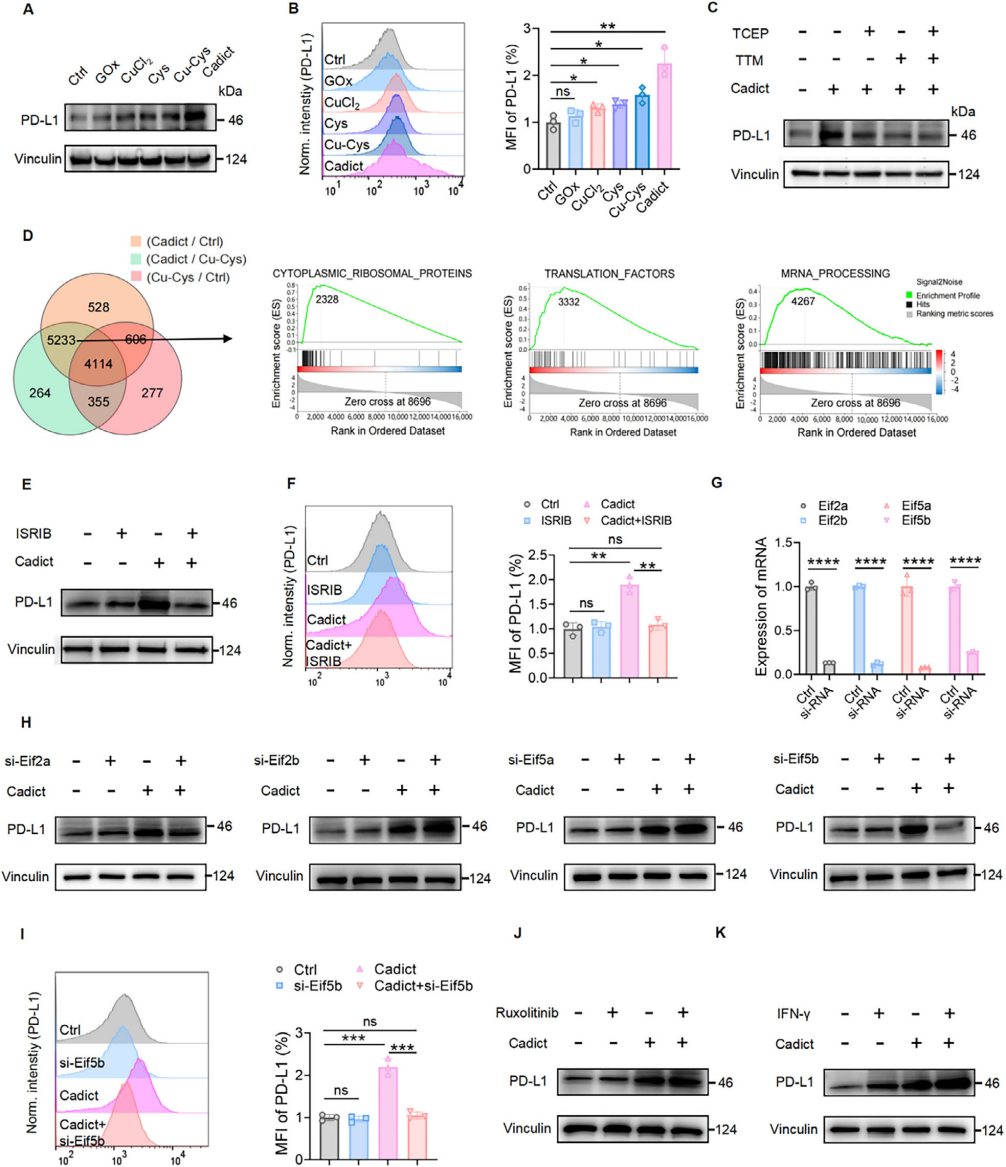
Cadict enhances PD‐L1 expression via Eif5b‐dependent translation. A) Western blot analysis of PD‐L1 expression in MC38 cells treated with: GOx, CuCl_2_, Cys, Cu‐Cys, or Cadict for 36 h. B) Flow cytometry profiles and quantification of PD‐L1 expression in treated MC38 cells (n = 3). C) Western blot analysis of PD‐L1 expression in MC38 cells pretreated with TTM (20 µm) or TCEP (1 mm) for 12 h, then exposed to Cadict for 36 h. D) RNA‐seq analysis of MC38 cells treated with PBS, Cu‐Cys, or Cadict (n = 3). Gene set enrichment pathway analysis (GSEA) showing significant enrichment in cytoplasmic ribosomal proteins, translation factors, and mRNA processing pathways in Cadict‐treated MC38 cells. E) Western blot analysis of PD‐L1 protein expression in MC38 cells treated with ISRIB (200 nm) or/and Cadict (2 µm) for 36 h. F) Flow cytometry profiles and quantification of PD‐L1 expression in MC38 cells treated with ISIRB or/and Cadict for 36 h (n = 3). G) qRT‐PCR analysis of relative mRNA expression in MC38 cells transfected with control or Eif2a, Eif2b, Eif5a, Eif5b siRNA (n = 3). H) Western blot analysis of PD‐L1 expression in MC38 cells after Eif2a/Eif2b/Eif5a/Eif5b knockdown and Cadict treatment (2 µm, 36 h). I) Flow cytometry profiles and quantification of PD‐L1 expression in Eif5b knockdown MC38 cells after Cadict treatment (2 µm) (n = 3). J, K) Western blot analysis of PD‐L1 expression in MC38 cells treated with Ruxolitinib (10 µm) (J), IFN‐γ (10 ng ml^−1^) (K), or/and Cadict (2 µm) for 36 h. Data are presented as mean ± SD, with one‐way ANOVA test (B, F, I) or unpaired two‐tailed Student's *t*‐test (G). ns, non‐significant, ^*^ *p* < 0.05, ^**^ *p* < 0.01, ^***^ *p* < 0.001, ^****^ *p* < 0.0001.

To investigate the mechanisms underlying Cadict‐induced PD‐L1 upregulation, we first compared PD‐L1 expression in CRC cells treated with Cadict or its individual components. Aligned with the synergistic effects of cuproptosis and disulfidptosis inducers, GOx, CuCl_2_, or Cu‐Cys monotherapies elevated PD‐L1 levels, with Cadict eliciting the greatest increase (Figure 6A,B). Blocking cuproptosis with TTM or disulfidptosis with TCEP effectively reversed Cadict‐triggered PD‐L1 upregulation, confirming the contribution of both cell death pathways (Figure 6C). Interestingly, Cadict did not alter PD‐L1 mRNA levels (Figure S5A), suggesting post‐transcriptional regulation. RNA‐sequencing analysis revealed that transcripts related to type II interferon (IFN‐γ) signaling were enriched in both Cu‐Cys‐ and Cadict‐ treated cells (Figure S5B). Importantly, Cadict‐treated cells demonstrated specific enrichment in cytoplasmic ribosomal proteins, translation factors, and mRNA processing pathways (Figure 6D), implicating a translational control mechanism. We thus assessed the stability of PD‐L1 mRNA and protein following Actinomycin D and cycloheximide (CHX) treatment, respectively, and observed no significant changes (Figure S5C,D). In response to stressors like viral infection, protein misfolding, or nutrient deprivation, mammalian cells modulate gene expression by suppressing global translation and preferentially translating specific mRNAs, a process known as the integrated stress response (ISR) [57, 58]. We then posited that Cadict facilitates PD‐L1 translation via the ISR pathway. Supporting this, inhibition of ISR with ISRIB completely mitigated Cadict‐induced PD‐L1 upregulation (Figure 6E,F). During ISR, phosphorylation of translation initiation factor eIF2α suppresses translation, yet alternative factors such as eIF2D and eIF5B can compensate under stress conditions [59, 60]. To pinpoint the specific factor involved, we individually knocked down Eif2a, Eif2b, Eif5a, and Eif5b, which were upregulated by Cadict (Figure 6G). Intriguingly, only Eif5b depletion strongly reduced PD‐L1 protein levels in Cadict‐treated MC38 cells, indicating its critical role in Cadict‐mediated PD‐L1 translation (Figure 6H,I; Figure S5E).

Given that tumor cells upregulate PD‐L1 in response to IFN‐γ stimulation [61], we further investigated whether IFN‐γ signaling contributed to Cadict‐induced PD‐L1 expression. Inhibition of IFN‐γ signaling with Ruxolitinib did not impair PD‐L1 upregulation by Cadict, while exogenous IFN‐γ further amplified it (Figure 6J,K). Collectively, these data demonstrate that Cadict enhances PD‐L1 expression primarily through Eif5b‐dependent translation, independent of IFN‐γ signaling.

### 2.6 Cadict Combined with Anti‐PD‐L1 Synergistically Enhances Antitumor Efficacy

**FIGURE 7:**
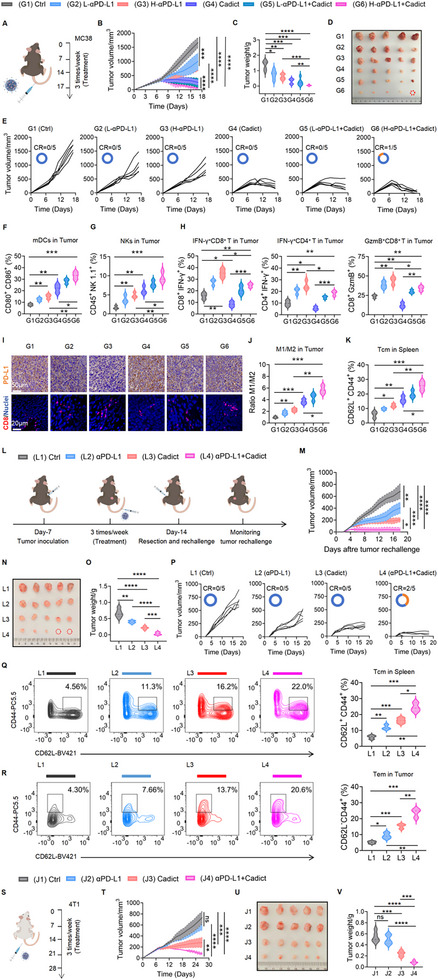
Cadict‐anti‐PD‐L1 combination demonstrates synergistic antitumor activity. A) Treatment schedule in the subcutaneous MC38 tumor model. B, E) Quantification of tumor volumes (B) and tumor growth kinetics (E) across treatment groups [complete response: CR] (n = 5). C, D) Representative images (C) and tumor weight statistics (D) of excised MC38 across treatments (G1, Ctrl; G2, L‐αPD‐L1; G3, H‐αPD‐L1; G4, Cadict; G5, L‐αPD‐L1+Cadict; G6, H‐αPD‐L1+Cadict) (n = 5). F, G) The percentages of matured DCs (CD80^+^CD86^+^) (F) and NK cells (CD45^+^NK1.1^+^CD3^−^) (G) in MC38 tumors across treatments (n = 3). H) The percentages of IFN‐γ^+^CD8^+^ T, IFN‐γ^+^CD4^+^ T, and GzmB^+^CD8^+^ T cells in MC38 tumors (n = 3). I) Representative images of IHC staining of PD‐L1 and IF staining of CD8^+^ T cells in the tumor sections. J) The M1/M2 macrophage ratios in MC38 tumors across treatments (n = 3). K) Quantification of splenic central memory T cells (Tcm, CD44^+^CD62L^+^) from MC38 tumor‐bearing mice (n = 3). L) Schematic illustration of rechallenge study design. M, P) Quantification of rechallenge tumor volumes (M) and growth kinetics (P) [complete response: CR] (n = 5). N, O) Representative images (N) and weight (O) of excised rechallenge tumors (n = 5). Q, R) Representative flow cytometry plots and quantification of splenic central memory T cells (Tcm, CD44^+^CD62L^+^) (Q), and tumor‐infiltrating effector memory T cells (Tem, CD44^+^CD62L^−^) (R) from MC38 tumor‐bearing mice (n = 3). S) Schematic of the subcutaneous 4T1 tumor treatment regimen. T) Tumor growth curves for each treatment group (n = 5). U, V) Representative images (U) and quantification of excised tumor weights (V) at study endpoint (n = 5). (J1, Ctrl; J2, αPD‐L1; J3, Cadict; J4, αPD‐L1+Cadict). Data are presented as mean ± SD, with two‐way ANOVA test (B, M, T) or one‐way ANOVA test (C, F‐H, J, K, O, Q, R, V). ns, non‐significant, ^*^ *p* < 0.05, ^**^ *p* < 0.01, ^***^ *p* < 0.001, ^****^ *p* < 0.0001.

Since Cadict‐induced intratumoral PD‐L1 upregulation could suppress T cell activity and limit antitumor efficacy, we hypothesized that combining Cadict with anti‐PD‐L1 blockade would synergistically enhance therapeutic outcomes. To test this, MC38 tumor‐bearing mice were treated with Cadict, low‐/high‐ dose of anti‐PD‐L1 (125 µg vs. 250 µg per mouse), or combination therapies (Figure 7A). Cadict monotherapy achieved stronger tumor suppression than either anti‐PD‐L1 dose alone. Although low‐ and high‐dose anti‐PD‐L1 treatments exhibited similar efficacy, combining Cadict with either dose further improved tumor control in a dose‐dependent manner, confirming synergistic effects (Figure 7B–E). Body weights remained stable across all groups (Figure S6A). The Cadict+high‐dose anti‐PD‐L1 cohort achieved the greatest tumor regression, with complete tumor eradication observed in 20% of mice (Figure 7E). Serum levels of proinflammatory cytokines (IL‐6, IFN‐γ, and TNF‐α), and IL‐2, a key driver of T cell proliferation, increased incrementally across treatments, paralleling therapeutic efficacy. Conversely, levels of anti‐inflammatory cytokine IL‐10 inversely correlated with tumor control (Figure S6B). Splenic DCs, NK, and T cells also expanded across treatment groups, with the most pronounced increase observed in the Cadict+high‐dose anti‐PD‐L1 cohort (Figure S6C–E).

Consistent with systemic immune activation, Cadict treatment enhanced mature DCs and NK cells infiltration within the TME (Figure 7F,G). However, it paradoxically reduced the proportions of CD4^+^/CD8^+^ T, IFN‐γ^+^CD4^+^ T, and GzmB^+^CD8^+^ T cells to less than half of control levels (Figure 7H), consistent with earlier observations (Figure 5). Intriguingly, anti‐PD‐L1 blockade reversed this T cell suppression, suggesting that checkpoint inhibition neutralizes Cadict‐induced intratumoral PD‐L1 upregulation (Figure 7I; Figure S6F). Moreover, MDSCs (CD11b^+^Gr‐1^+^) infiltration was reduced across treatment groups, while the M1/M2 polarization ratio of tumor associated macrophages (TAMs) increased post‐treatments, the effects of which were most robust in the Cadict+high‐dose αPD‐L1 group (Figure 7J; Figure S6G). Memory T cells play a pivotal role in sustaining long‐term antitumor immunity and preventing relapses. Analysis of splenic central memory T cells (Tcm, CD44^+^CD62L^+^) revealed that Cadict+high‐dose anti‐PD‐L1 treatment induced the greatest proportions of Tcm (Figure 7K; Figure S6H). To further assess durable antitumor responses, we tested the therapeutic efficacy of Cadict combined with anti‐PD‐L1 blockade with tumor rechallenge model. After primary tumors in MC38 tumor‐bearing mice were resected following treatment with Cadict, anti‐PD‐L1 blockade, or their combination, the same MC38 cells were reinjected on the contralateral flank to monitor tumor outgrowth (Figure 7L). Cadict monotherapy exhibited significantly stronger antitumor efficacy than anti‐PD‐L1 monotherapy, while the combination treatment showed the most potent and synergistic tumor control (Figure 7M–O). Notably, in the Cadict+anti‐PD‐L1 group, 40% of mice achieved complete tumor eradication (Figure 7P). To investigate the underlying immunological memory, we analyzed splenic Tcm (CD44^+^CD62L^+^) and tumor‐infiltrating effector memory T cells (Tem, CD44^+^CD62L^−^). The results demonstrated that Cadict+anti‐PD‐L1 treatment induced the highest proportions of both Tcm and Tem subsets (Figure 7Q,R). Collectively, these data clearly indicate that Cadict synergizes with anti‐PD‐L1 therapy to elicit robust and durable immunological memory, thereby establishing a foundation for long‐term antitumor immunity.

Finally, to test broad applicability in a more stringent, immunologically cold setting, we assessed Cadict efficacy in the triple‐negative 4T1 breast cancer model—characterized by low baseline PD‐L1 and poor T‐cell infiltration. The therapeutic efficacy observed in the 4T1 model was consistent with and extended our previous findings in the CT26 model. Anti‐PD‐L1 monotherapy showed only minimal activity against these resistant tumors, whereas Cadict alone induced significant tumor growth inhibition. Most importantly, the combination of Cadict with anti‐PD‐L1 therapy resulted in markedly superior antitumor efficacy, effectively overcoming the intrinsic resistance of this immunologically refractory model (Figure 7S–V; Figure S6I, J). Altogether, these results demonstrate that Cadict synergizes with anti‐PD‐L1 to remodel the immunosuppressive TME, strengthen antitumor immunity, and establish durable immunological memory, supporting its broad therapeutic potential in immunologically cold or checkpoint‐refractory tumors.

## 3 Discussion

Tumor immunotherapy faces two major challenges: immune checkpoint inhibitor resistance and immunosuppressive TME [62, 63]. Emerging “dual death induction” strategies employ nanomaterials to simultaneously trigger two complementary cell death pathways, overcoming limitations of single‐pathway therapies while boosting tumor elimination and immune activation. For example, coupling ferroptosis with other immunogenic death pathways (e.g., pyroptosis or necroptosis) amplifies anti‐tumor immunity [64, 65, 66], and its combination with cuproptosis or disulfidptosis selectively disrupts cancer cell redox homeostasis [67, 68, 69, 70]. However, the therapeutic potential of co‐inducing cuproptosis and disulfidptosis remains underexplored. More critically, the unclear interplay between these death pathways and their immunomodulatory effects impedes the development of novel therapies.

Inspired by our finding that dual induction of cuproptosis and disulfidptosis synergistically enhances tumor cytotoxicity and anti‐PD‐L1 sensitivity, we developed Cadict—an EGFR‐targeted nanotherapeutic that co‐delivers inducers of both cell death pathways. This multifunctional system amplifies cytotoxic effect and anti‐tumor immune activation to maximize therapeutic efficacy. Cadict displayed high tumor‐targeting specificity and favorable biosafety profiles in vivo. By selectively initiating dual lethal programs in cancer cells, it promoted the release of DAMPs and pro‐inflammatory cytokines, triggering systemic immune activation and reprogramming the immunosuppressive TME. Moreover, Cadict upregulated PD‐L1 expressions in tumor cells, sensitizing them to anti‐PD‐L1 therapy and creating therapeutic vulnerability.

The Cadict nanoplatform is constructed from cystine, S‐Cu‐S quasi‐planar structures, and GOx, encapsulating Cu (II) and Cu (I) ions at a 1:2 molar ratio. The Cys‐Cu coordination represents a classic nano‐coordination system first characterized in 1997 and subsequently explored for enzymatic, fluorescent, and radiosensitizing properties [44, 45, 46]. Preclinical evaluations in murine and lapine models have confirmed its therapeutic efficacy and safety, supporting its clinical translational potential [71]. Surface functionalization with a GE11 peptide enables EGFR‐targeted delivery and intracellular drug release via endocytosis‐mediated lysosomal fusion. Intracellularly released copper ions selectively accumulate in tumor cells, inducing cuproptosis, while GOx‐mediated glucose depletion synergizes with cystine accumulation to promote disulfidptosis under hypoglycemic conditions. Cadict exhibits favorable physicochemical properties, including a hydrodynamic diameter of 11.7 nm, a PDI of 0.294, 6‐day colloidal stability, and an in vivo pharmacokinetics half‐life (t_1/2_) of 1.22 h. Collectively, these features highlight the rational design of Cadict, leveraging a clinically translatable Cys‐Cu platform for tumor‐specific co‐delivery of cuproptosis and disulfidptosis inducers. Paradoxically, although NAC is widely recognized as a protector against disulfidptosis via disulfide exchange [72], it can also act as a Cu(II)‐dependent pro‐oxidant, inducing ROS‐mediated cytotoxicity [73]. In our experimental system, Cadict serves as a copper source, which likely shifts the dominant role of NAC toward its pro‐oxidant function. Thus, although NAC may initially alleviate disulfidptosis, its interaction with Cadict‐released Cu(II) forms a potent redox‐active complex that ultimately promotes ROS‐mediated cell death. Consistent with this mechanism, we observed in additional experiments that increasing NAC concentrations correlated with elevated cell mortality in Cadict‐treated MC38 cells (Figure S4R), supporting the copper‐dependent pro‐oxidant role of NAC [74].

ICB targeting the PD‐1/PD‐L1 axis has transformed cancer therapy, yet elucidating tumor‐intrinsic and ‐extrinsic mechanisms modulating PD‐L1 expression remains a major barrier to improving clinical outcomes. PD‐L1 regulation is multifaceted, spanning genetic [75], transcriptional [76, 77, 78], post‐transcriptional [79], and post‐translational levels [80, 81]. While copper ions are known to upregulate PD‐L1 through HIF‐1α‐ and NF‐κB‐mediated transcription or via cGAS‐STING signaling [82, 83, 84], how different cell death pathways‐especially when combined‐ modulate PD‐L1 expression remains unclear. Here, we uncovered an unexpected synergy between cuproptosis and disulfidptosis in boosting PD‐L1 expression in CRC cells through Eif5b‐mediated translational upregulation, bypassing the canonical IFN‐γ‐dependent transcriptional control [61], and sensitizing CRC to anti‐PD‐L1 therapy. Our data demonstrates that Cadict‐induced PD‐L1 translation depends on ISR activation, pinpointing a critical link between cellular stress adaptation and immune checkpoint regulation. The ISR is a conserved adaptive mechanism deployed by eukaryotic cells to counteract proteotoxic stress, nutrient deprivation, pathogen invasion, and oxidative damage [85]. In this context, Cadict depletes intracellular GSH and glucose, triggering excess H_2_O_2_ production, NADPH exhaustion, cystine accumulation, and redox collapse, culminating in ISR activation. While eIF2α phosphorylation is the canonical hallmark of ISR, we identified Eif5b as an alternative translation initiator driving PD‐L1 upregulation, consistent with prior reports [86]. This divergence underscores the plasticity of ISR signaling, wherein the nature and intensity of stress dictate cellular outcomes [87].

While this engineered nanoplatform pioneers a therapeutic paradigm integrating redox‐metabolic dysregulation with immunomodulation, several important challenges necessitate focused attention. First, maintaining sufficient intracellular copper levels is critical for sustaining cuproptosis induction in vivo. Second, further optimization in clinically relevant models is imperative to validate therapeutic efficacy, safety, and applicability across diverse cancer types and disease stages. Third, delineating the mechanistic crosstalk between cuproptosis and disulfidptosis within the TME, such as their effects on immune cell functionality, cytokine networks, and metabolic rewiring, will be critical to refine therapeutic synergy and mitigate off‐target toxicity. Nevertheless, this study establishes a compelling foundation for Cadict as a next‐generation platform that couples dual cell death induction with immune remodeling. Together, these findings position Cadict as a promising strategy to overcome immune resistance and enhance the efficacy of cancer immunotherapy.

## 4 Conclusion

This study develops Cadict, an EGFR‐targeted nanoplatform that co‐induces cuproptosis and disulfidptosis via coordinated delivery of copper ions, GOx, and cystine. Beyond eliciting potent tumor cytotoxicity, manifesting features of ICD, and enhancing antitumor immunity, Cadict activates the integrated stress response, leading to Eif5b‐mediated PD‐L1 upregulation. This dual action synergistically sensitizes tumors to anti‐PD‐L1 therapy, resulting in robust tumor suppression and durable immunological memory. Our work thereby establishes a novel strategy that leverages tumor redox vulnerabilities to overcome intrinsic resistance in low PD‐L1‐expressing tumors and enhance the efficacy of cancer immunotherapy.

## 5 Methods

### 5.1 Materials

Elesclomol (ES, #STA‐4783), BAY‐876 (#S8452), Ferrostatin‐1 (Fer‐1, #S7243), Chloroquine (CQ, #S4157), Ruxolitinib (#S1378), ISRIB (#S0706) were from Selleck (USA). Necrostatin‐1 (Nec‐1, #HY‐15760), DPQ (#HY‐114869), Pepstatin (#HY‐P0018), L‐NAME (#HY‐18729A), N‐acetyl cysteine (NAC, #HY‐B0215), and BOC‐D‐FMK (#HY‐13229) were from MedChem Express (MCE, USA). Tris (2‐chloroethyl) phosphate (TCEP, #ST045), Recombinant mouse IFN‐γ protein (#RP01070), and Tetrathiomolybdate (TTM, #323446), CuCl_2_ (#203149), GOx (#G7141), Cystine (Cys, #779539) were from Beyotime, Abclonal, and Sigma–Aldrich, respectively. DSPE‐PEG‐GE11 (#909204‐1), Actinomycin D (#HY‐17559), and Cycloheximide (CHX, #HY‐112951) were obtained from MeloPEG. All reagents were dissolved according to the manufacturer's instructions.

### 5.2 Cell Culture

Mouse colorectal cancer cells MC38 (CVCL_B288), CT26 (CVCL_7254) and breast cancer cells 4T1 (CVCL_0125) were obtained from the American Type Culture Collection (ATCC, Manassas, VA, USA), which were cultured at 37(C in a humidified incubator containing 5% CO_2_ with RPMI 1640 medium supplemented with 10% fetal bovine serum (FBS), 100 U ml^−1^ penicillin/streptomycin and 1 mm sodium pyruvate following CTCC guidelines. All cell lines were routinely examined using the Short Tandem Repeat (STR) method, and the results of the mycoplasma test were negative.

### 5.3 Co‐Culture

CD8^+^ T cells were extracted from the splenic tissue of C57BL/6 mice and followed by centrifugation, then the T cells were seeded into a 24‐well plate with RPMI medium containing 10% FBS and 1% penicillin/streptomycin. Recombinant IL‐2 protein (Proteintech, #1015), CD3 Recombinant antibody (Proteintech, #81324), and CD28 Recombinant antibody (Proteintech, #65099) were supplemented for CD8^+^ T cell maturation. The medium was half‐replaced every 2 days. On the fifth day of culture, microscopically observed that the T cells were well differentiated and transferred to a new 24‐well plate for co‐culture with MC38 cells (T cell: Tumor cell = 1: 1). Subsequently, PBS, ES (10 nm) + CuCl_2_ (1 µm) + BAY‐876 (10 nm), αPD‐L1(10 µg ml^−1^), ES + CuCl_2_ + BAY‐876 + αPD‐L1 were added to co‐culture system to treat tumor cells for 24 h, respectively. Finally, the cells were stained and measured with a flow cytometer (Attune NxT, Thermo Fisher) and analyzed with FlowJo software, version 10.8.1.

### 5.4 Preparation and Character of Cadict Nanodrugs

The Cuproptosis and Disulfidptosis co‐delivery targeted (Cadict) nanodrug was synthesized via a coordination self‐assembly method. Briefly, 25 mm Copper was dissolved in the 7 mL ultrapure water, 25 mm cystine dissolved in 1 mL of HCl (1 m), 10 mg of GOx was dissolved in 1 mL ultrapure water, and 2 mg of DSPE‐mPEG‐GE11 was dissolved in 1 mL ultrapure water. The GOx solution was then added to the copper solution, followed by dropwise addition of the cystine solution into the mixture. Finally, the DSPE‐mPEG‐GE11 was rapidly added. The final solution was stirred at room temperature for 4 h. Subsequently, the mixture was concentrated by ultrafiltration at 3000 ×g for 30 min (10 kDa ultrafiltration tube obtained from Merck Millipore). The Zetasizer Nano ZS instrument (Malvern Instruments Ltd., UK) was used to measure particle size distribution, polydispersity index (PDI), and zeta potential. Transmission electron microscopy (Thermo Fisher Scientific, Japan) and high‐resolution transmission electron microscopy (TECNAI G2, FEI, Maastricht, Netherlands) were employed to characterize the morphology of Cys‐Cu. The crystallographic properties were analyzed by XPS and XRD methods. The copper ion concentration was determined by ICP‐MS (Agilent 7700, USA), and the encapsulation efficiency of GOx was quantified using the Coomassie Brilliant Blue method.

### 5.5 In Vivo Imaging of Biodistribution

To evaluate the targeting performance of Cadict in mice, the MC38 tumor‐bearing mice were intravenously injected with ICG or Cadict@ICG, and biodistribution images were collected by In Vivo Imaging System (PerkinElmer/IVIS Lumina III) at 0, 2, 4, 6, 12, 24, and 48 h. For the ex vivo biodistribution study, a subset of mice was sacrificed 12 h after injection treatment. Ex vivo imaging of major organs, including the heart, liver, spleen, lung, kidney, and tumor, was collected and analyzed quantitatively with the IVIS spectrum imaging system.

### 5.6 In Vitro Cellular Uptake of Cadict

First, Cadict loaded ICG, and then analyzed intracellular Cadict@ICG uptake by CLSM and FCM. For CLSM observation, MC38 cells were seeded on 15 mm Glass‐bottomed cell culture dishes (Nest, #801002) and incubated at 37°C for 12 h. Then, the cells were incubated with Cadict@ICG or ICG (control) for 6 h. Subsequently, the cell culture medium was removed and washed with PBS for three times, followed by incubation with LysoTracker Green DND‐26 (CST, #8783S) for 30 min. After incubation, the cells were stained for the nuclei of cell with mounting medium of antifading with DAPI (Solarbio, #S2110‐5). Finally, images were acquired with CLSM. For FCM detection, MC38 cells were seeded into a 6‐well plate at 5 × 10^5^ cells per well and incubated at 37°C for 12 h. The Cadict and a GE11‐unconjugated DSPE‐mPEG (labeled with the ICG fluorophore) were added to wells, respectively. Afterward, the cells were harvested at 2, 4, 6, 12, and 24 h, respectively. Finally, the cells were measured with a CytoFLEX flow cytometer (Beckman Coulter) and analyzed in detail using FlowJo software, version 10.8.1.

### 5.7 Mitochondrial Morphology Observation

MC38 cells were seeded at a density of 1 × 10^6^ per well in 6‐well plates for 12 h. Subsequently, the cells were treated with PBS or Cadict (2 µm) for 24 h. Then, the cells were collected, fixed with electron microscope fixative (Basmedtsci, #BP0130), and observed by Bio‐TEM.

### 5.8 Pharmacokinetic Assay of Cadict

After intravenous injection of Cadict (5 mg kg^−1^) into C57BL/6 mice (n = 3), 5–10 µl blood was collected at 0, 1, 2, 4, 6, 12, and 24 h. Subsequently, the blood samples were digested and diluted, and the concentration of Cu in the blood samples was measured by Agilent ICP‐MS.

### 5.9 Cell Viability and Colony Formation Assay

For the cell viability assay, 1 × 10^4^ cells were seeded into a 96‐well plate and treated with the indicated chemicals. Cell viability was measured using the Cell Counting Kit‐8 (MCE, #HY‐K0301) according to the manufacturer's instructions.

For the colony‐forming assay, 1 × 10^3^ cells were seeded in a 6‐well plate and treated continuously with chemical drugs. The colonies were fixed using 4% formaldehyde, stained with a 0.1% solution of crystal violet in water, and counted with the Image J v1.53c software.

### 5.10 Cell Migration Assay

For the assessment of the migration of cells, the assay was performed using polycarbonate membrane chambers with a pore size of 8 µm (Corning, #3422). 1.0 × 10^5^ cells were seeded into the upper transwell chamber with 200 µl of serum‐free medium. The tumor cells were treated with PBS, GOx (0.2 µg ml^−1^), CuCl_2_ (0.2 µg ml^−1^), Cys (0.2 µg ml^−1^), Cu‐Cys (2 µm), Cadict (2 µm) for 24 h. The group of cells to which PBS was added was used as a negative control. The lower chamber was filled with 600 µl medium containing 20% FBS as a chemoattractant. After a 24 h migration, cells that migrated to the lower side were fixed by using 4% paraformaldehyde for 30 min, and stained with 0.1% crystal violet for another 30 min, subsequently photographed with a microscope, and counted by image analysis software. The results of the experiment were determined by three replications for precision.

### 5.11 Live/Dead Cell Staining Analysis

MC38 cells were cultured in a 6‐well culture plate (5 × 10^5^ cells per well) and incubated at 37°C with complete medium for 12 h. Then the cells were treated with PBS, GOx (0.2 µg ml^−1^), CuCl_2_ (0.2 µg ml^−1^), Cys (0.2 µg ml^−1^), Cu‐Cys (2 µm), Cadict (2 µm) for 24 h. Subsequently, the cells were stained with working solution (2 µm Calcein AM and 8 µm PI) at 37°C for 30 min in the dark. Labeled cells were observed under a fluorescence microscope. Calcein AM‐labeled live cells (Ex/Em: 495 nm/520 nm) fluoresced green, and PI‐labeled dead cells (Ex/Em: 530/620 nm) fluoresced red.

### 5.12 Measurement of Reactive Oxygen Species (ROS) Production

Dichlorodihydrofluorescein diacetate (DCFH‐DA) was used as a fluorescent probe to detect the level of intracellular ROS. In brief, MC38 cells (5 × 10^5^ per well) were seeded on 6‐well plates in 1 mL complete medium and incubated at 37°C for 12 h. The cells were treated with PBS, GOx (0.2 µg ml^−1^), CuCl_2_ (0.2 µg ml^−1^), Cys (0.2 µg ml^−1^), Cu‐Cys (2 µm), Cadict (2 µm) for 24 h respectively. Subsequently, the intracellular ROS of harvested cells were detected by FCM.

### 5.13 Apoptosis Analysis

To determine cellular apoptosis levels, cells were assessed with Annexin V‐FITC/PI Apoptosis Detection Kit (4Abio, #FXP018‐100). MC38 cells were seeded on 6‐well plates at 5 × 10^5^ cells per well. After 12 h incubation, the tumor cells were treated with PBS, GOx (0.2 µg ml^−1^), CuCl_2_ (0.2 µg ml^−1^), Cys (0.2 µg ml^−1^), Cu‐Cys (2 µm), Cadict (2 µm) for 24 h, washed with PBS, and incubated with Annexin/PI reagent in the dark for 10 min at 25°C. Thereafter, the cells were immediately measured with a CytoFLEX flow cytometer (Beckman Coulter) and analyzed in detail using FlowJo software, version 10.8.1.

### 5.14 Detection of Intracellular GSH

MC38 cells were seeded into 6‐well plates and incubated for 12 h. Tumor cells were treated with PBS, GOx (1 µg ml^−1^), CuCl_2_ (1 µg ml^−1^), Cys (1 µg ml^−1^), Cu‐Cys (10 µm), and Cadict (10 µm) for 6 h, respectively. After incubation, the cells were washed three times with PBS and dissociated with trypsin. Cells were collected and then detected with Reduced Glutathione (GSH) Content Assay Kit (Solarbio, #BC1170).

### 5.15 Detection of Intracellular NADP^+^/NADPH Ratio

MC38 cells in RPMI complete medium were seeded in 6‐well plates, and incubated for 12 h. After that, the cells were treated with PBS, GOx (1 µg ml^−1^), CuCl_2_ (1 µg ml^−1^), Cys (1 µg ml^−1^), Cu‐Cys (10 µm), Cadict (10 µm) for 6 h. Subsequently, the cells were washed three times with PBS and dissociated with trypsin. Finally, the cells were collected and then detected by CoenzymeII NADP(H) Content Assay Kit (Solarbio, #BC1100).

### 5.16 Western Blot

Cells were lysed by utilizing RIPA buffer (Beyotime, #P0013B), followed by centrifugation of the resulting lysates at 12 000 rpm for 30 min at 4°C. After that, the supernatants were collected, and their protein levels were measured with a BCA protein assay kit (Beyotime, #P0011). Cytoplasmic and nuclear extracts were separated from CRC cells with NE‐PER Nuclear and Cytoplasmic Extraction Reagents (Thermo Scientific, #78833). The lysates underwent SDS‐PAGE and were subsequently transferred onto PVDF membranes (Merk Millipore, #IPVH00010), followed by immunoblotting with the primary antibodies such as GAPDH (Proteintech, #10494), Vinculin (Abclonal, #A2752), PD‐L1 (Proteintech, #66248), LIAS (Proteintech, #11577), EIF5B (Proteintech, #13527), FDX1 (Abcam, #108257), and DLAT (CST, #12362S). Secondary antibodies conjugated to Horseradish peroxidase, either anti‐rabbit/mouse IgG, aided in the detection process. The signal intensity was quantified using the ImageJ software.

### 5.17 Plasmid Construction and Transfection

For siRNA‐mediated Eif2a, Eif2b, Eif5a, and Eif5b silencing, MC38 cells were transfected with siRNA duplex and Lipofectamine RNAiMAX (Invitrogen, #13778075) according to the manufacturer's instructions. A nonspecific siRNA oligo (Sigma, #SIC002) was used as a negative control. The sequences of siRNA were listed as: Eif2a (CACTATAAGCAAGTCTCTTAA), Eif2b (GTAGAGAACGGAGGCATTA), Eif5a (GCTGGACTCCTATTCAAAT), Eif5b (GTGGAGCTGAAGAAAGTAT).

### 5.18 RNA Isolation, qRT‐PCR

The FastPure Cell/Tissue Total RNA Isolation Kit (Vazyme, #RC112) was used to isolate total RNA according to the manufacturer's instructions, and the total RNA was used to synthesize cDNA by the *Evo M‐MLV* RT Kit (Accurate Biology, #AG11706). Real‐time PCR was performed on a QuantStudio 5 F (Applied Biosystem) using the Premix Pro Taq HS qPCR Kit II (Accurate Biology, #AG11702). Target gene expression was normalized to that of the control gene GADPH abundance. The qRT‐PCR primers were synthesized by TSINGKE biological technology and their sequences listed as: GAPDH (F: CATCACTGCCACCCAGAAGACTG, R: ATGCCAGTGAG CTTCCCGTTCAG), PD‐L1 (F:GCTCCAAAGGACTTGTACGTG, R: TGATCTG AAGGGCAGCATTTC), Eif2a (F: CTGGGACGCCTAACCTACAAC, R: TCCGG GCACAAATAATTTCAT), Eif2b (F: GGTTGAGTGGAAAGAGGCGTA, R: TGG ATGCTGCTGTCGGCTATA), Eif5a (F: AAGGTCCATCTGGTTGGCATTG,R: TGTCCTGGAGCAGGGATAGGT), Eif5b (F: GGGTTTGAATGCTGCTTTGTT, R: AACCTCCATGACTTGTGCTCT).

### 5.19 RNA Sequencing (RNA‐seq) and Analysis

Total RNAs from MC38 cells (Ctrl vs. Cu‐Cys vs. Cadict) were extracted with TRIzol reagent (Invitrogen, #15596026) and sequenced on the Illumina Novaseq 6000 sequencing instrument (BGI, China). The DESeq package was used to analyze differential genes with the conditions of Fold Change ≥2 and adjusted *p* value ≤ 0.001. The R programming language was used to draw a heatmap of differential gene clusters, and GSEA was performed using GSEA software according to related methods (PMID: 16199517). Classifying differentially expressed genes according to the GO and KEGG annotation results and classifications, and analyzed the KEGG enrichment pathway using phyper in the R software, While GO enrichment pathway was analyzed by the TermFinder package. Candidate genes were defined as significantly enriched under the condition with a Q value of ≤0.05 as the threshold.

### 5.20 Animal Experiments

BALB/c and C57BL/6 mice (female, 4 to 6 weeks old, 16 to 20 g) were obtained from Guangdong GemPharmatech (Guangdong, China). All animal procedures were conducted in accordance with the National Institute of Health Guide for the Care and Use of Laboratory Animals. The experimental procedures followed an approved protocol from the Institutional Animal Care and Use Committee of Sun Yat‐sen University (approval numbers: SYSU‐IACUC‐2023002381, SYSU‐IACUC‐2024003438).

1.0 × 10^6^ MC38 (CT26) cells were subcutaneously injected into C57BL/6 (BALB/c) mice. The mice were split into four categories: PBS, ES (5 mg kg^−1^) +CuCl_2_ (2 mg kg^−1^) +BAY‐876 (3 mg kg^−1^), αPD‐L1 (250 µg per mouse), ES+CuCl_2_+BAY‐876+αPD‐L1. The therapies were started on day 7 post‐tumor inoculation, and intraperitoneal (ES+CuCl_2_+BAY‐876) or intravenous (αPD‐L1) injections were administered every 3 days. Tumor volume was measured regularly every 2 days since day 7 of tumor implantation and calculated using the formula V = L × W^2^/2 (V, volume, L, length, and W, width). The C57BL/6 and BALB/c mice were sacrificed on day 21, and the tumors were photographed, weighed, analyzed by flow cytometry, and embedded in paraffin for further pathological analysis.

To evaluate the pharmacological safety of Cadict and GOx, an experiment was conducted to set up different concentrations of Cadict (1, 3, 5, 7, and 9 mg kg^−1^) and GOx (3 mg kg^−1^) intravenously injected into MC38 tumor‐bearing mice every 3 days since day 7 post‐tumor inoculation. The body weight, tumor weight, and volume of the mice were measured regularly.

To evaluate the antitumor efficacy of chemical drugs, the MC38 tumor models were established by subcutaneously injecting 1 × 10^6^ tumor cells on the flank of C57BL/6 mice. The tumor bearing mice were randomly divided into Six groups on day 7 post tumor inoculation, including: PBS, GOx (3 mg kg^−1^), CuCl_2_ (5 mg kg^−1^), Cys (5 mg kg^−1^), Cu‐Cys (5 mg kg^−1^), and Cadict (5 mg kg^−1^), which were intravenously injected with different formulations in 100 µl of sterile PBS every 3 days for five times.

For Cadict and αPD‐L1 monotherapy or combination therapy, mice bearing MC38 or 4T1 tumors received intravenous injections every 3 days beginning on day 7 post‐inoculation (1 × 10^6^ per mouse). Treatment consisted of Cadict (5 mg kg^−1^) and/or 125 µg per mouse αPD‐L1 (Low dose group) or 250 µg per mouse αPD‐L1 (High dose group), or their combination. MC38‐bearing mice received 4 doses, and 4T1‐bearing mice received 6 doses.

To establish a tumor rechallenge model, primary MC38 tumors were resected from mice on day 14 post‐inoculation, following treatment with Cadict (5 mg kg^−1^), αPD‐L1 (250 µg per mouse), or their combination. The same number of MC38 cells (1 × 10^6^) was then injected into the contralateral flank to assess tumor development.

### 5.21 In Vivo Biosafety Evaluation

Female C57BL/6 mice were randomly divided into six groups, with 6 mice in each group. Each mouse was injected with a single dose of PBS, GOx (3 mg kg^−1^), CuCl_2_ (5 mg kg^−1^), Cys (5 mg kg^−1^), Cu‐Cys (5 mg kg^−1^), and Cadict (5 mg kg^−1^), respectively. The mice were monitored and weighed every 2 days. Then the mice were sacrificed at 13 days after administration, and the mice's blood samples were collected for serum biochemical analysis. Blood samples were centrifuged at 3000 rpm for 10 min to collect plasma, which was further used for analysis of alanine transaminase (ALT), aspartate aminotransferase (AST), blood urea nitrogen (BUN), UREA, and lactate dehydrogenase (LDH). Furthermore, the main organs (heart, liver, spleen, lung, and kidney) were dissected from the mice for analysis after being sacrificed. All the tissues were paraffin‐embedded, and tissue sections were prepared. Tissues were stained with Hematoxylin and Eosin (H&E) to observe pathological features, and images were captured using a digital whole slide imaging scanner (KF‐PRO‐020).

### 5.22 Hemolytic Activity Assay of Cadict

The hemolytic activity of Cadict was evaluated using mouse red blood cells (RBCs). Briefly, 100 µl of a suspension containing 1 × 10^6^ RBCs was mixed with diluted test solutions and incubated at 37°C for 30 min. PBS and deionized water were used as negative and positive controls, respectively. Additionally, RBC suspensions were treated with varying concentrations of Cadict for 6 h. Subsequently, the mixtures were centrifuged at 1500 rpm for 10 min. Photographs of the supernatants were captured to assess hemoglobin release, indicative of RBC lysis.

### 5.23 Immunohistochemistry (IHC)

IHC assays were conducted utilizing standard methods. Briefly, tissue sections from paraffin‐embedded mouse colorectal cancer specimens were deparaffinized in xylene and rehydrated with graded ethanol. Post rehydration, the sections were stained with primary antibodies with anti‐Ki67 (Abcam, #ab92742) and anti‐PD‐L1 (Proteintech, #66248). Following the primary antibody application, and then treated with secondary antibody and visualized with DAB Detection Kit (ZSGB‐BIO, #ZLI‐9017).

IHC staining analysis was performed by two independent observers who were blinded to the results of this experiment. The images were acquired with a KF‐PRO‐020 scanner (Konfoong Tech), and the quantification of staining was accomplished with ImageJ software. The staining intensity score was categorized into four levels: 0 (no staining), 1+ (weak staining), 2+ (moderate staining), 3+ (strong staining). The H‐scores in tumor tissues were calculated by multiplying the intensity of staining by the extent of reactivity, yielding a potential range from 0 to 300.

### 5.24 Immunofluorescent (IF)

For CRC cells, the cells were seeded on 15 mm glass‐bottomed cell culture dishes (Nest, #801002), fixed with 4% paraformaldehyde (PFA) for 15 min, permeabilized with 0.5% Triton X‐100 in PBS (PBTX) for 15 min at room temperature, followed by blocking and incubated for 12 h with primary antibodies such as DLAT (1:500, CST, #12362S), F‐actin(1:500, Abcam, #ab205), HMGB1 (1:400, Proteintech, #10829), Calreticulin (1:200, Proteintech, #27298). Subsequently, secondary antibody Alexa Fluor 555‐labeled Donkey Anti‐Rabbit IgG (1:500, Beyotime, #A0453) or Alexa Fluor 488‐labeled Goat Anti‐Mouse IgG (1:500, Beyotime, #A0428) was incubated with cells for 1 h in the dark. And then stained the nuclei of cell with mounting medium of antifading with DAPI (Solarbio, #S2110‐5). Finally, images were captured by an Olympus FV3000 Laser Scanning Confocal Microscope and evaluated using FV31S‐SW software.

For CRC tissue, a tissue apoptosis assay was performed using a Fluorometric TUNEL assay kit (Promega, #G3250). Immunofluorescent staining for tumor tissue was performed under the same conditions as the cells processing described above. Primary antibodies include: DLAT (1:100), F‐actin (1:100), CD8 (1:250, BioLegend, #100708), HMGB1 (1:100), Calreticulin (1:100). However, unlike cell processing, some tissue incubation with fluorescently labeled primary antibody did not require secondary antibody.

### 5.25 Flow Cytometry Analysis

The mice were euthanized at the indicated time points. The samples, including tumor, spleen, and blood, were collected, and then tumors were subjected to enzymatic digestion using RPMI medium supplemented with 10% FBS, collagenase type IV (1 mg ml^−1^; Invitrogen, #17104019), and deoxyribonuclease (20 U ml^−1^, Roche, #10104159001) at 37°C for 1 h. Washed the suspension with PBS and filtered through a 70 µm strainer. The cells were centrifuged and subsequently utilized for FACS analysis.

For CD4^+^ T, CD8^+^ T, NK, Th1, Th2, Tregs, Tcm, Tem, and PD‐L1 protein analysis, the cells were stained with anti‐mouse CD45 (BioLegend, #103112, #103139), anti‐mouse CD3 (BioLegend, #100222, #100236), anti‐mouse CD4 (BioLegend, #100422, #100406), anti‐mouse CD8 (BioLegend, #100708, #100706), anti‐mouse IFN‐γ (BioLegend, #505808), and anti‐human/mouse Granzyme B (BioLegend, #396407). anti‐mouse NK‐1.1 (BioLegend, #108706), anti‐mouse IL4 (BioLegend, #504118), anti‐mouse CD25 (BioLegend, #102005), anti‐mouse FOXP3 (ThermoFisher, #12‐5773‐80), anti‐mouse CD62L (BioLegend, #104436), anti‐mouse/human CD44 (BioLegend, #103032), anti‐mouse CD274 (BioLegend, #124311). For mature DCs, the cells were stained with anti‐mouse CD45 (BioLegend, #103140), anti‐mouse CD11c (BioLegend, #117318), anti‐mouse I‐A/I‐E (BioLegend, #107607), anti‐mouse CD80 (BioLegend, #104705), and anti‐mouse CD86 (BioLegend, #105114). For M1 and M2 macrophages, MDCSs, the cells were stained with anti‐mouse CD45 (BioLegend, #103140), anti‐mouse/human CD11b (BioLegend, #101206), anti‐mouse Gr‐1 (BioLegend, #108407), anti‐mouse F4/80 (BioLegend, #123114), anti‐mouse CD86 (BioLegend, #105029), and anti‐mouse CD206 (BioLegend, #141708). All the cells were stained with the antibodies for 30 min at 4°C. To stain intracellular proteins, the cell suspension was fixed and permeabilized with the Immunostaining Permeabilization Buffer with Triton X‐100 (Beyotime, #P0095). Flow cytometry was performed (Attune NxT, Thermo Fisher) according to its standard operating procedures, and the data were analyzed using FlowJo v10.8.1 software.

### 5.26 Statistical Analysis

All in vitro experiments were independently repeated at least three times, and the animal experiments were replicated at least twice. The number of biological replicates (n) is indicated in the figure legends. Unless otherwise stated, all quantitative data are presented as individual data points with the mean ± SD. All statistical analyses were performed using GraphPad Prism 9. A two‐tailed Student's *t*‐test was used for comparisons between two groups. One‐way or two‐way analysis of variance (ANOVA) was used to analyze multiple groups (≥3). The statistical methods are specified in the figure legends, with statistical significance set at p < 0.05. Data collection and analysis for animal experiments and IHC staining were conducted by researchers blinded to group allocation.

## Author Contributions

Conceptualization, M.S.; Data analysis and Curation, S.H., S.S., X.Z.; J.G., R.P., J.D., X.W., L.C., Q.L., X.L.; Investigation and Validation, W.H., M.S., Q.Z., X.Z.; Writing – Original Draft, M.S., S.H, S.S.; Writing – Review& Editing, W.H., M.S., Q.Z.; Supervision and Funding acquisition, W.H., M.S.

## Conflicts of Interest

The authors declare no conflicts of interest.

## Supporting information




**Supporting File**: advs74531‐sup‐0001‐SuppMat.docx.

## Data Availability

RNA‐seq data that support the findings of this study have been deposited in the Gene Expression Omnibus with accession code GSE304102. All other data supporting the findings of this study are available from the corresponding authors on reasonable request.
