# Supplementary material for: Cuproptosis and Disulfidptosis Converge to Empower PD‐L1 Checkpoint Therapy via Cadict‐Induced PD‐L1 Translation
Source: Adv Sci (Weinh). 2026 Feb 21;13(25):e15367. doi: 10.1002/advs.202515367 (PMC13137794; doi:10.1002/advs.202515367)
Supplement: Supplementary file 1 — Supporting File: advs74531‐sup‐0001‐SuppMat.docx. [file ADVS-13-e15367-s001.docx]

Supporting Information for

**Cuproptosis and disulfidptosis converge to empower PD-L1 checkpoint therapy via Cadict-induced PD-L1 translation**

*Shaoqing Huang^#^, Shiyao Song^#^, Xinhua Zhang^#^, Jialing Gao, Ruihan Pu, Jiatong Dai, Xiaoxue Wu, Lulu Chen, Qinghai Li, Xiaoting Liu, Qi Zhou*, Mei Song*, Weiling He**

S. Huang, X. Zhang, Q. Li, M. Song, W. He

Center for Gastrointestinal Surgery, The First Affiliated Hospital, Sun Yat-sen University, Guangzhou, Guangdong, 510080, China.

Email: [songm7@mail.sysu.edu.cn](mailto:songm7@mail.sysu.edu.cn)

Email: wlhe@xah.xmu.edu.cn

S. Song, J. Dai

Department of Pediatrics, The First Affiliated Hospital, Sun Yat-sen University, Guangzhou, Guangdong, 510080, China.

J. Gao, X. Wu, L. Chen, M. Song

Institute of Precision Medicine, The First Affiliated Hospital, Sun Yat-Sen University, Guangzhou, Guangdong, 510275, China

R. Pu

School of Public Health, Sun Yat-sen University, Guangzhou, Guangdong, 510275, China

X. Liu

Department of Immunology, Zhongshan School of Medicine, Sun Yat-sen University, Guangzhou, Guangdong, 510080, China.

S. Huang, Q. Zhou

Center for Hepato-Pancreatico-Biliary Surgery, The First Affiliated Hospital, Sun Yat-sen University, Guangzhou, Guangdong, 510080, China.

Email: zhouqi@mail.sysu.edu.cn

Q. Zhou

Department of General Surgery, Hui Ya Hospital of The First Affiliated Hospital, Sun Yat-sen University, Huizhou, Guangdong, 516081, China.

W. He

Department of Gastrointestinal Surgery, Xiang'an Hospital of Xiamen University, School of Medicine, Xiamen University, Xiamen, Fujian, 361000, China.

**Supporting Figures**

**
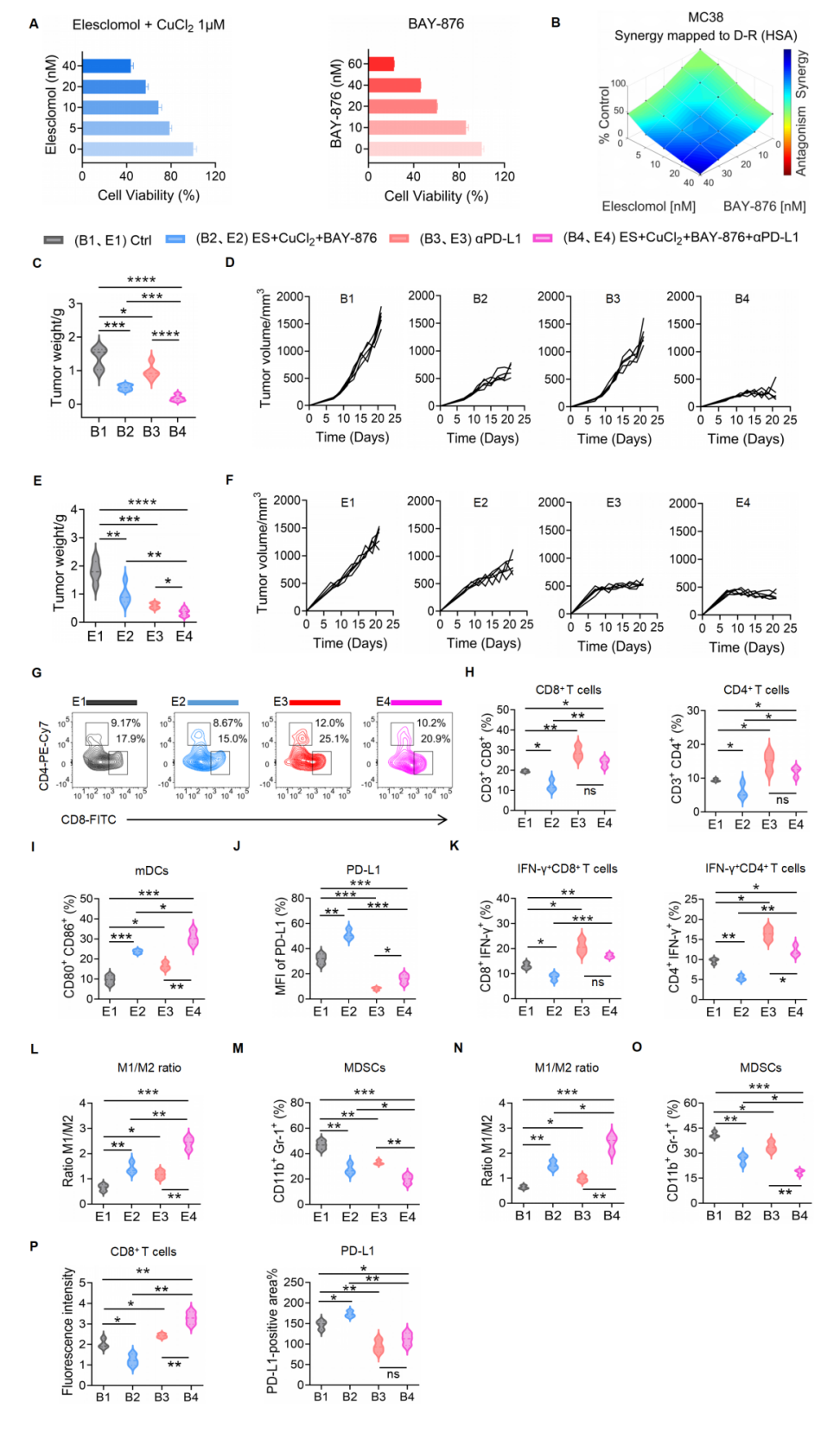
**

**Figure S1.** Cuproptosis-disulfidptosis synergy potentiates anti-tumor immunity and ICB efficacy. A) Viability of MC38 cells treated with Elesclomol (ES-0, 5, 10, 20, 40 nm) + CuCl_2_(1 μm) or BAY-876 (0, 10, 20, 40, 60 nm). B) Clonogenic survival of MC38 cells treated with vehicle, ES+CuCl_2_, BAY-876, or their combination (n=3 independent experiments). Colonies were quantified after 10 days (ImageJ v1.53c). Drug synergy was analyzed using Combenefit (blue: synergy; red: antagonism). C, D) Quantification of tumor weight (C) and growth curves (D) in CT26-bearing mice across treatment groups (B1, Ctrl; B2, ES+CuCl_2_+BAY-876; B3, αPD-L1; B4, ES+CuCl_2_+BAY-876+αPD-L1) (n=5). E, F) Quantification of tumor weights (E) and growth curves (F) in MC38-bearing mice across treatment groups (E1, Ctrl; E2, ES+CuCl_2_+BAY-876; E3, αPD-L1; E4, ES+CuCl_2_+BAY-876+αPD-L1) (n=5). G, H) Representative flow cytometry plots (G) and quantification (H) of tumor infiltrating CD3^+^CD8^+^ and CD3^+^CD4^+^ T cells in MC38 tumors (n=3). I) The percentages of mature DCs (CD80^+^CD86^+^) populations in MC38 tumors (n=3). J) Quantification of PD-L1 expression in treated MC38 tumors (n=3). K) The percentages of IFN-γ^+^CD8^+^, IFN-γ^+^CD4^+^ T cell populations in MC38 tumors (n=3). L, N) M1/M2 macrophage polarization ratios in MC38 (L) and CT26 (N) tumors across treatment groups (n=3). M, O) The percentages of tumor infiltrating MDSCs (CD11b^+^ Gr-1^+^) in MC38 (M) and CT26 (O) tumors across treatment groups (n=3). P) Quantification of CD8^+^ T and PD-L1 expression in treated CT26 tumors (n=3). Data are presented as mean ± SD, with one-way ANOVA test (C, E, H-P). ns, non-significant, **p* < 0.05, ***p* < 0.01, ****p* < 0.001, *****p* < 0.0001.


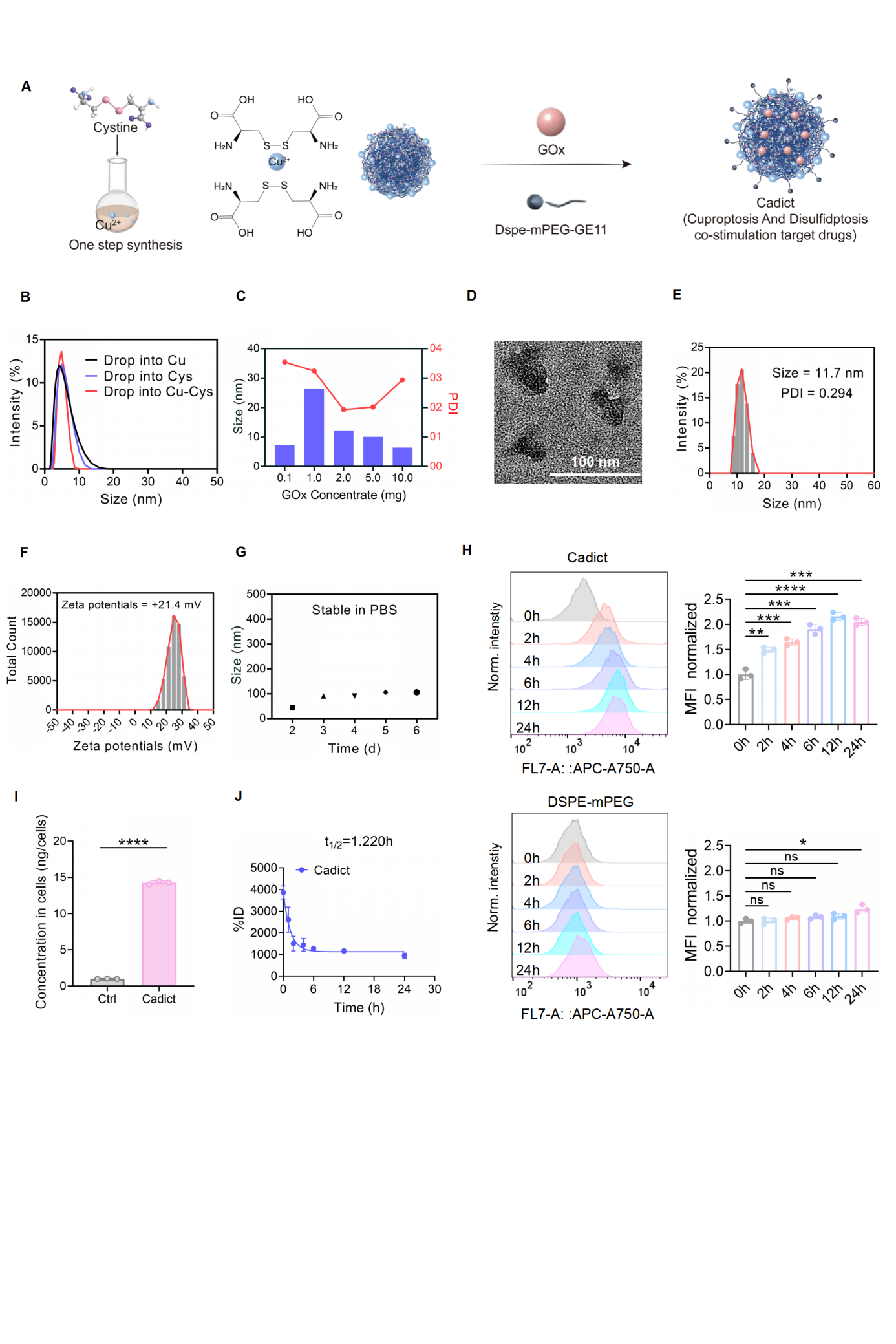


**Figure S2.** Synthesis and characterization of Cadict. A) Schematic diagram of Cadict synthesis process. B) Effect of drop order on Cadict particle size. C) Effect of GOx dosage on Cadict particle size. D) Representative TEM image of Cadict. Scale bar: 100 nm. E, F) Particle size distribution (E) and Zeta potentials (F) of Cadict measured by DLS. G) Stability of Cadict in PBS solution for 6 days. H) Flow cytometry analysis of intracellular accumulation over time for Cadict and the non-targeted control (DSPE-mPEG loaded with ICG) in MC38 cells. I) The copper concentration in MC38 cells after incubation with Cadict for 4 h. J) The pharmacokinetics assessment of copper after *i.v.* injection of Cadict. Data are presented as mean ± SD, with one-way ANOVA test (H) or unpaired two-tailed student's t-test (I). ns, non-significant, **p* < 0.05, ***p* < 0.01, ****p* < 0.001, *****p* < 0.0001.


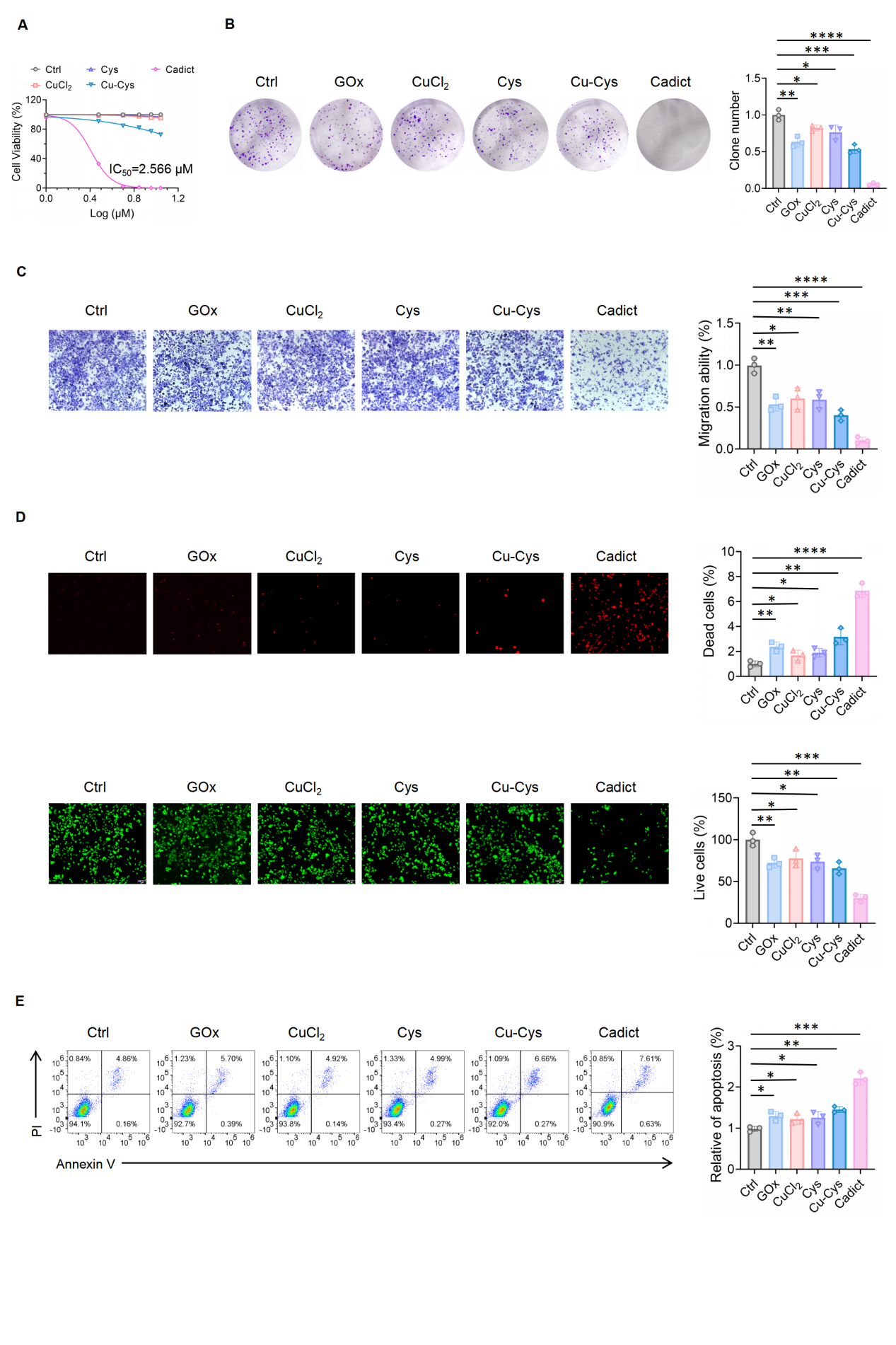


**Figure S3.** Cadict exhibits superior cytotoxicity in tumor cells. A) Cadict cytotoxicity in MC38 cells: IC_50_ =2.566 μm (24 h). B) Representative images and quantitative analysis of MC38 colony formation across treatments (PBS, GOx, CuCl_2_, Cys, Cu-Cys, Cadict) (n=3). C) Representative images and quantitative analysis of MC38 transwell assay across treatments (n=3). D) Representative images and quantification of Live/dead staining in treated MC38 cells (n=3). E) Representative images and quantification of apoptosis rates in MC38 cells across treatments (n=3). Data are presented as mean ± SD, with one-way ANOVA test (B-D). **p* < 0.05, ***p* < 0.01, ****p* < 0.001, *****p* < 0.0001.


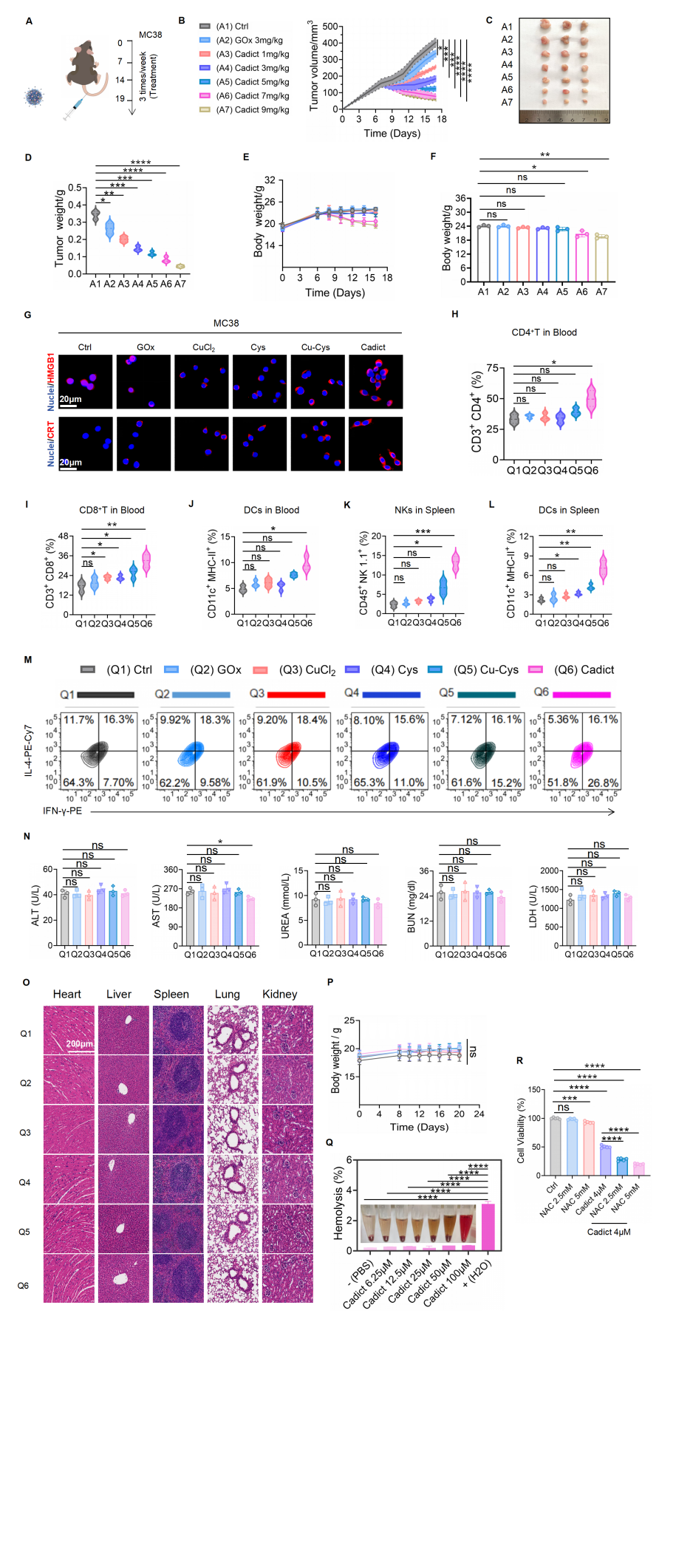


**Figure S4.** Cadict elicits ICD-like effects and activates antitumor immunity *in vivo*. A) Dosing schedule of Cadict in MC38 tumor model. B) Quantification of MC38 tumor volumes (n=3). C, D) Representative images (C) and tumor weight statistics (D) of excised MC38 tumors across treatments (A1, Ctrl; A2, GOx, 3mg kg^-1^; A3, Cadict, 1mg kg^-1^; A4, Cadict, 3mg kg^-1^; A5, Cadict, 5mg kg^-1^; A6, Cadict, 7mg kg^-1^; A7, Cadict, 9mg kg^-1^) (n=3). E, F) Quantitative analysis of body weights of MC38-bearing mice (n=3). G) Representative immunofluorescence images of HMGB1 and CRT in MC38 cells treated with PBS, GOx (0.2 μg ml^-1^), CuCl_2_ (0.2 μg ml^-1^), Cys (0.2 μg ml^-1^), Cu-Cys (2 μm), Cadict (2 μm) for 12 h. H-J) The percentages of serum CD3^+^CD4^+^ T, CD3^+^CD8^+^ T cells (H, I) and DC cells (CD11c^+^MHC-II^+^) (J) from MC38-bearing mice (Q1, Ctrl; Q2, GOx; Q3, CuCl_2_; Q4, Cys; Q5, Cu-Cys; Q6, Cadict) (n=3). K, L) Splenic NK cells (CD45^+^NK1.1^+^CD3^-^, K) and DC cells (CD11c^+^MHC-II^+^, L) from MC38-bearing mice (n=3). M) Representative flow cytometry plots of tumor-infiltrating Th1(IFN-γ⁺) and Th2(IL-4^+^) cells from MC38 -bearing mice (n=3). N) Serum biochemistry in treated MC38-bearing mice (n=3): ALT, AST, UREA, BUN, LDH. O) H&E staining of major organs (heart, liver, spleen, lung, kidney). P) Body weight changes in treated MC38-bearing mice (n=6). Q) Hemolysis assay of mouse RBCs by Cadict (6 h), with positive control: H_2_O, and negative control: PBS (n=3). R) Viability of MC38 cells pretreated with NAC (2.5 and 5 mm) for 12 h, followed by Cadict (2 μm) for 24 h. Data are presented as mean ± SD, with two-way ANOVA test (B, P) or one-way ANOVA test (D, F, H-L, N, Q, R). ns, non-significant, **p* < 0.05, ***p* < 0.01, ****p* < 0.001, *****p* < 0.0001.


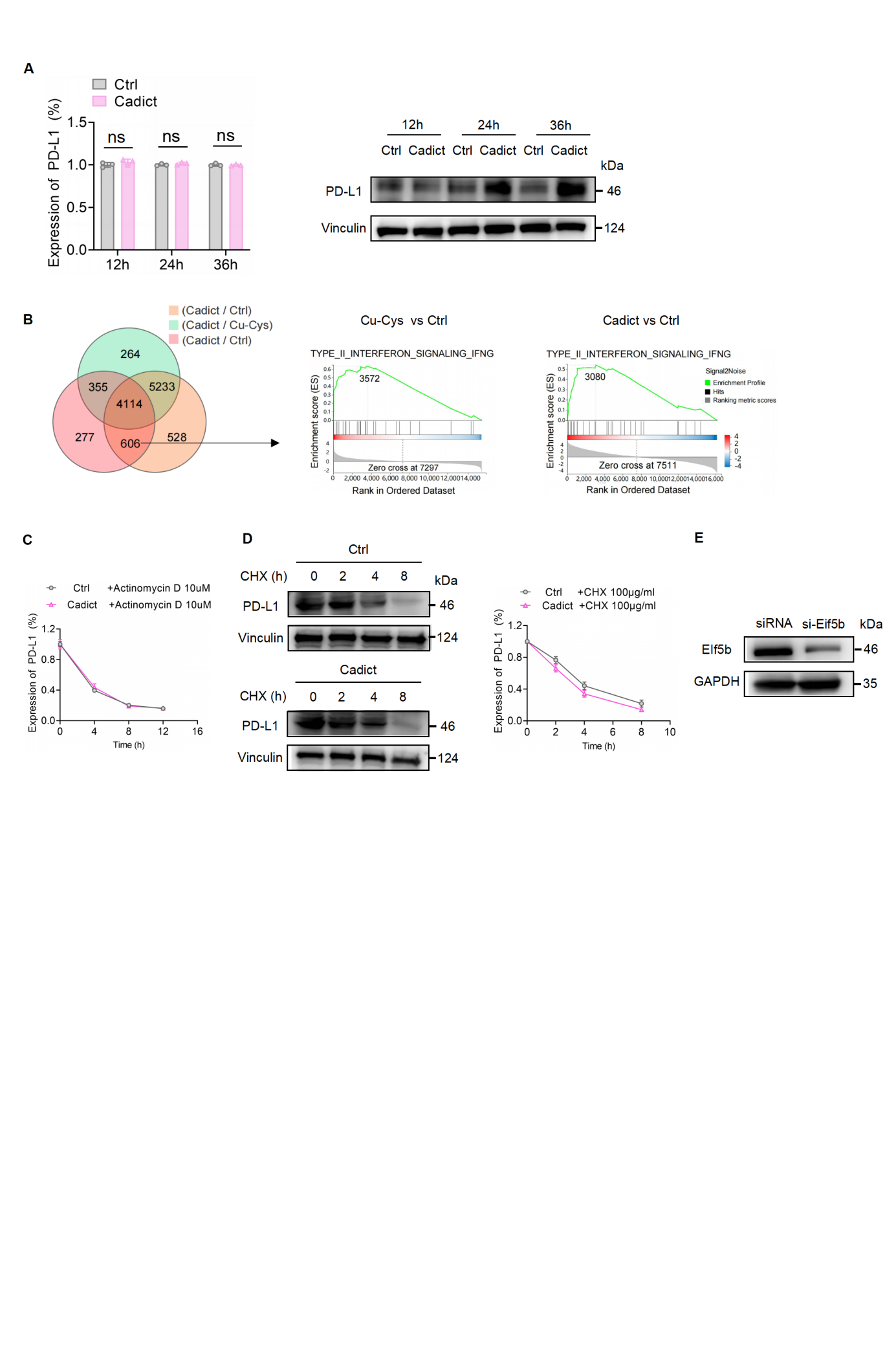


**Figure S5.** Cadict upregulates PD-L1 expression via Eif5b-mediated translation. A) qRT-PCR and western blot analysis of PD-L1 mRNA/protein expression in MC38 cells treated with Cadict (2 μm) at 12 h, 24 h, and 36 h (n=3). B) RNA-seq analysis of MC38 cells treated with PBS, Cu-Cys, or Cadict (n=3). Gene set enrichment pathway analysis (GSEA) showing significant enrichment of type Ⅱ interferon singling (IFN-γ) in both Cu-Cys and Cadict treated MC38 cells. C) qRT-PCR analysis of PD-L1 mRNA decay in MC38 after treatment with Actinomycin D (10 μm) and Cadict (2 μm) across four time points (n=3). D) Western blot quantification of PD-L1 protein stability after CHX (100 μg ml^-1^) treatment at indicated timepoints. Data were normalized to Vinculin (n=3 independent experiments). E) Western blot analysis of Eif5b protein expression in siRNA-transfected MC38 cells. Data are presented as mean ± SD, with two-way ANOVA test (A). ns, non-significant.


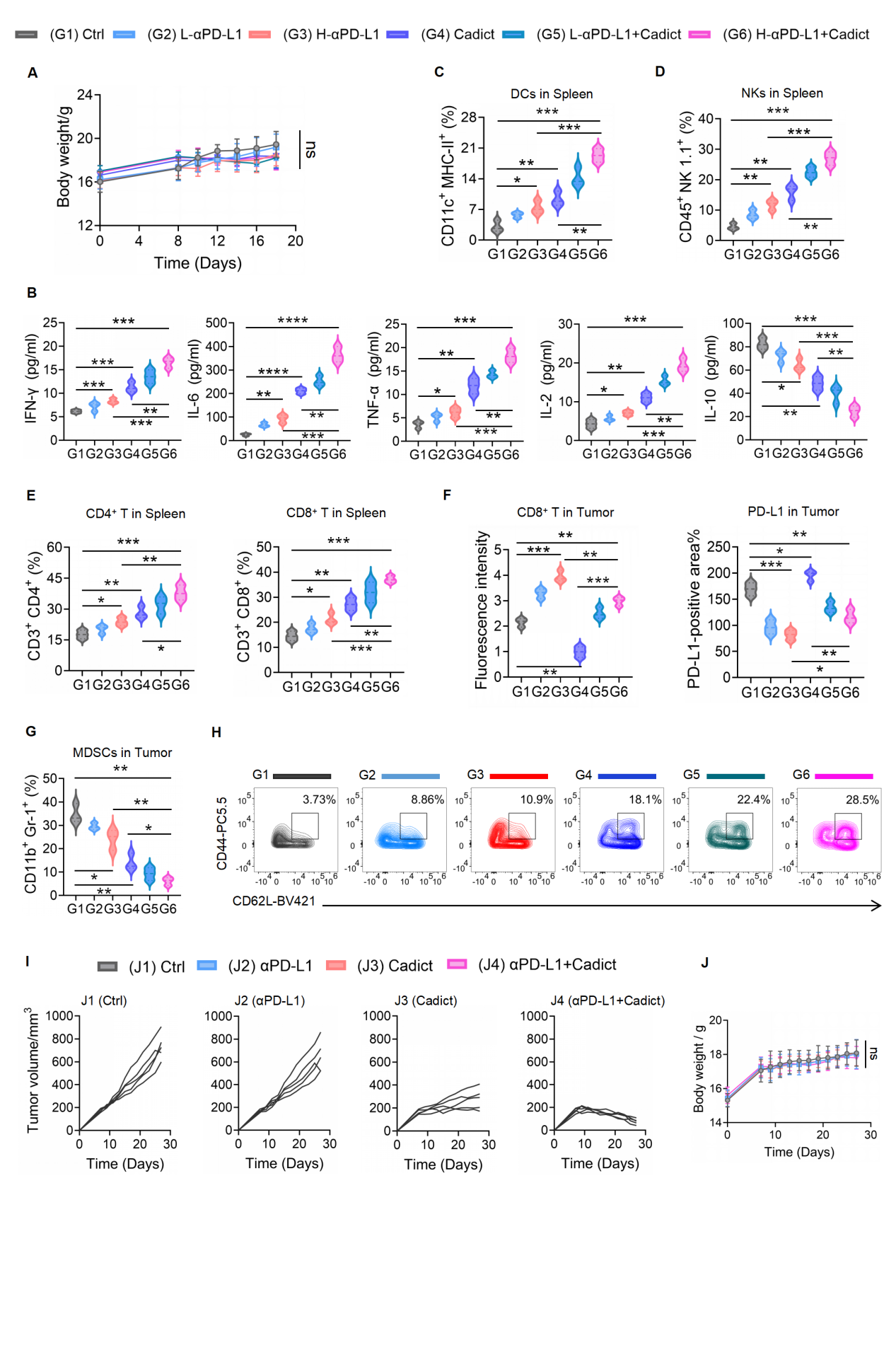


**Figure S6.** Cadict and anti-PD-L1 combination synergistically enhances antitumor activity. A) Quantitative analysis of body weight of MC38-bearing mice (n=5). B) Serum IFN-γ, IL-6, TNF-α, IL-2, and IL-10 levels in MC38 tumor bearing mice with different treatments (G1, Ctrl; G2, L-αPD-L1; G3, H-αPD-L1; G4, Cadict; G5, L-αPD-L1+Cadict; G6, H-αPD-L1+Cadict) (n=5). C-E) The percentages of DC cells (CD11c^+^MHC-II^+^) (C), NK cells (CD45^+^NK1.1^+^CD3^-^) (D), CD3^+^CD8^+^ T and CD3^+^CD4^+^ T cells (E) in the spleen of MC38 tumor-bearing mice (n=3). F) Quantification of CD8^+^ T and PD-L1 expression in treated MC38 tumors (n=3). G) The percentages of MDSCs (CD11b^+^Gr-1^+^) in MC38 tumors across treatments (n=3). H) Representative flow cytometry plots of splenic central memory T cells (Tcm, CD44^+^CD62L^+^) from MC38 tumor-bearing mice (n=3). I) 4T1 tumor growth kinetics across treatment groups (J1, Ctrl; J2, αPD-L1; J3, Cadict; J4, αPD-L1+Cadict) (n=5). (**J**) Body weight changes in 4T1-bearing mice over the treatment peroid (n=5). Data are presented as mean ± SD, with two-way ANOVA test (A, J) or one-way ANOVA test (B-G). ns, non-significant, **p* < 0.05, ***p* < 0.01, ****p* < 0.001, *****p* < 0.0001.

**
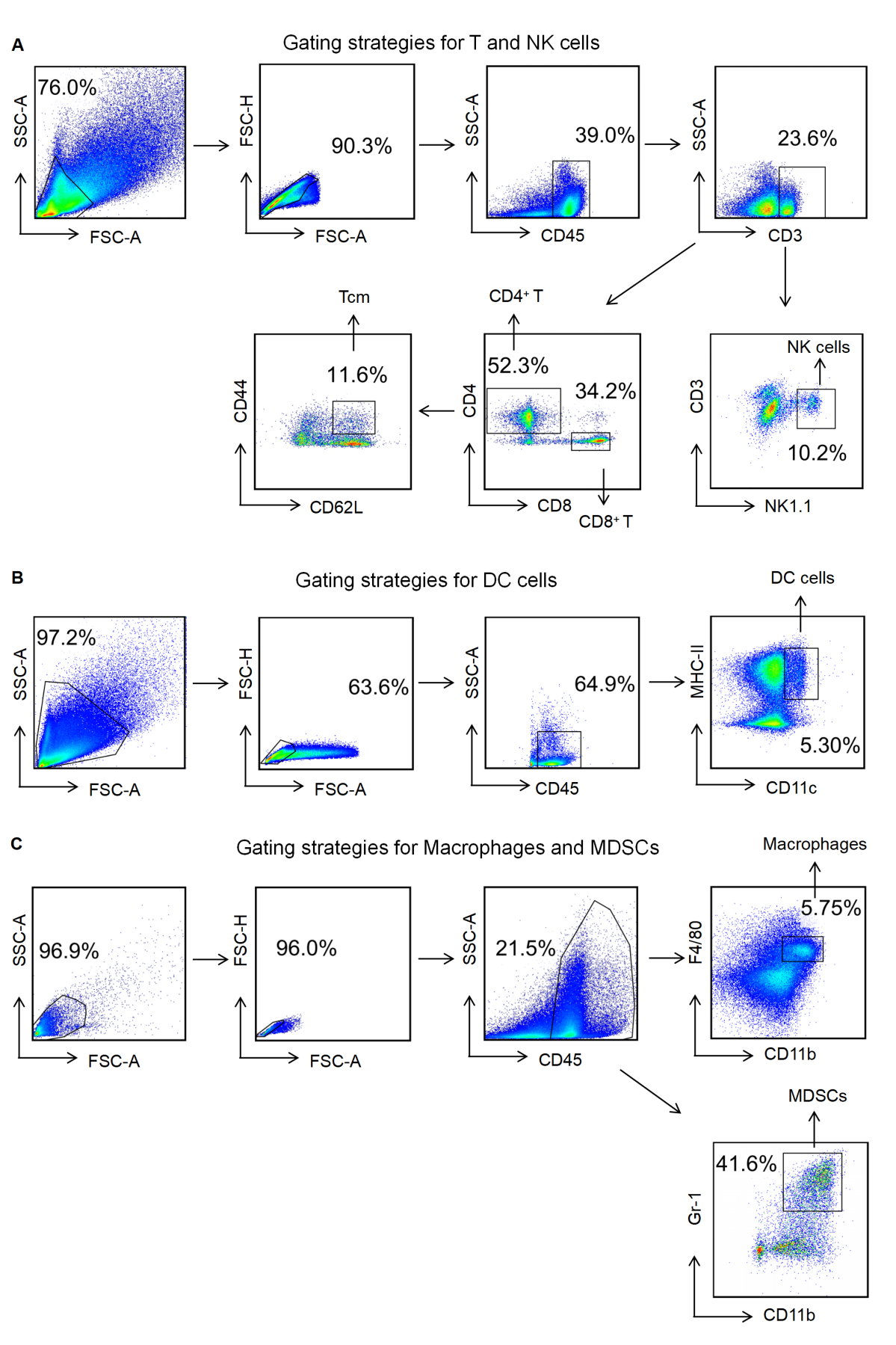
**

**Figure S7.** Gating strategies for flow cytometry analysis. A) Gating strategy for (Figure 2J, N, Q), (Figure 5K-N, Q), (Figure 6G, H, K, Q, R), (Figure S1H, K), (Figure S4H, I, K, M), (Figure S6D, E, H). B) Gating strategy for (Figure 2L), (Figure. 5I, J), (Figure 6F), (Figure S1I), (Figure S4J, L), (Figure S6C). C) Gating strategy for (Figure 5O, P), (Figure 7J), (Figure S1L-O), (Figure S6G).
